# A Systematic Narration of Some Key Concepts and Procedures in Plant Breeding

**DOI:** 10.3389/fpls.2021.724517

**Published:** 2021-09-16

**Authors:** Weikai Yan

**Affiliations:** Ottawa Research and Development Center, Agriculture and Agri-Food Canada (AAFC), Ottawa, ON, Canada

**Keywords:** heritability, genotype by environment interaction, optimum testing, optimum replication, multi-trait selection, biplot analysis, mega-environment analysis, breeder's equation

## Abstract

The goal of a plant breeding program is to develop new cultivars of a crop kind with improved yield and quality for a target region and end-use. Improved yield across locations and years means better adaptation to the climatic, soil, and management conditions in the target region. Improved or maintained quality renders and adds value to the improved yield. Both yield and quality must be considered simultaneously, which constitutes the greatest challenge to successful cultivar development. Cultivar development consists of two stages: the development of a promising breeding population and the selection of the best genotypes out of it. A complete breeder's equation was presented to cover both stages, which consists of three key parameters for a trait of interest: the population mean (μ), the population variability (σ_*G*_), and the achieved heritability (*h*^2^ or *H*), under the multi-location, multi-year framework. Population development is to maximize μσ_*G*_ and progeny selection is to improve *H*. Approaches to improve *H* include identifying and utilizing repeatable genotype by environment interaction (GE) through mega-environment analysis, accommodating unrepeatable GE through adequate testing, and reducing experimental error via replication and spatial analysis. Related concepts and procedures were critically reviewed, including GGE (genotypic main effect plus genotype by environment interaction) biplot analysis, GGE + GGL (genotypic main effect plus genotype by location interaction) biplot analysis, LG (location-grouping) biplot analysis, stability analysis, spatial analysis, adequate testing, and optimum replication. Selection on multiple traits includes independent culling and index selection, for the latter GYT (genotype by yield^*^trait) biplot analysis was recommended. Genomic selection may provide an alternative and potentially more effective approach in all these aspects. Efforts were made to organize and comment on these concepts and procedures in a systematic manner.

## Introduction

Plant breeding plays a key role in meeting the human needs for more food, nutrition, and fiber under a changing climate. The goal of a plant breeding program is to develop new cultivars of a crop kind with improved yield and quality for its target region and end-use. All theories, concepts, processes, procedures, and analyses related to plant breeding are developed and implemented around this goal. A target region is the target population of environments, which is the sum of soil, climatic, biotic, and abiotic conditions plus common management practices that are likely to be encountered in the region. Improved yield means improved adaptation to the target region, which is reflected in improved mean performance and stability of performance across locations and years in the target region. Improved quality means improved adaptation to the end-uses that bring value and income to the growers in the target region. Both the target environments and the target end-uses may change over time, in a predictable or unpredictable manner. Yield is the result from integrating numerous traits including various yield components, agronomic traits, disease resistances, and tolerance to various abiotic stresses characteristic of the target region. Consequently, yield in different regions may mean different ways of packaging these traits and underlying gene alleles. Likewise, quality is a collective term of many parameters for a specific end-use. Thus, dealing with many traits simultaneously is an essential task of cultivar development, although most breeding-related publications deal with only a single trait, typically yield. The relation between yield and other traits is analogous to that between the skin and the hair of a fur or that between the trunk and the branches of a tree; other traits gain importance only when attached to (i.e., combined with) high yield (Yan and Frégeau-Reid, 2008; Yan et al., 2019a). Plant breeding is a mature discipline of applied sciences, with well-developed concepts and procedures. Nevertheless, a systematic combing and narration of the numerous, sometimes confusing, concepts and procedures should help both new and experienced breeders in their work toward developing superior cultivars. The concepts and procedures in plant breeding are indeed much easier to tackle for a single trait. So, much of the discussion will be on a single trait while keep in mind that multi-trait selection is essential to cultivar development, which is discussed in the last section. In addition, genomic selection (Goddard and Hayes, 2007; Heffner et al., 2009; Jannink et al., 2010) has become a growing point or integral part in most plant breeding programs. Its role will be briefly mentioned when the various concepts and procedures are discussed.

## The Complete Breeder's Equation

The cultivar development process includes two stages: the development of a promising breeding population and the identification of the best progeny out of it. Breeding success can be measured by the following equation, referred to as the Complete Breeder's Equation (modified from Yan et al., 2019b),

(1) $$\begin{array}{l} \;B=\left(\mu +{ih\sigma }_{G}\right)/\left(YC\right), \end{array}$$

in comparison with the well-known Breeder's Equation of Eberhart (1970),

(2) $$\begin{array}{l} {\Delta G=ih\sigma }_{G}\text{/Y.} \end{array}$$

Here *B* stands for breeding success per unit time and cost and Δ*G* stands for selection gain over the population mean per unit time, for a trait of interest (typically yield). μ is the mean of the breeding population, σ_*G*_ is the square root of the genotypic variance of the population, *i* is the selection intensity in the unit of σ_*G*_, *h* is the square root of achieved heritability (*h*^2^ or *H*), *Y* is the length of the breeding cycle in years, and *C* is the operation cost per year. μ, σ_*G*_ *and h* are to be estimated from environments representing the target region. A target region may consist of multiple mega-environments, as will be discussed later. For the time being the target region is assumed to be a single mega-environment. A mega-environment is defined as a group of environments that share the same best cultivar(s) (Gauch and Zobel, 1997; Yan et al., 2000).

Relative to Equation 2, Equation 1 emphasizes the importance of population mean in cultivar development and serves as a reminder that any selection progress is on the basis of the population mean. The inclusion of *C* emphasizes that cultivar development is an enterprise that must consider the cost for the achieved genetic gain.

Cultivar development consists of two stages: population development and progeny selection. Practical breeders would agree that developing a promising breeding population, i.e., making a promising cross or crosses, is the crucial first step toward cultivar development. A promising breeding population is the basis for any meaningful selection effort. This point may be implied in Eberhart (1970) and by later researchers (e.g., Cobb et al., 2019; Rutkoski, 2019) when discussing the Breeder's Equation but its importance to cultivar development can never be overemphasized, thus implicitly indicated in Equation 1. The potential of a breeding population for cultivar development, shorted as population potential (*P*), depends on both the population mean (μ) and the population variability (σ_*G*_):

(3) $$\begin{array}{l} P=\sqrt{\mu \sigma_{G}}. \end{array}$$

Apparently, if there is no genetic variability, there would be no selection progress; if the population mean is low, it is unlikely to lead to any superior cultivars regardless of selection strategies. Practical plant breeders are well aware of the importance of the population mean. They cross best with best and look for recombinants better than both parents (Duvick, 1996). A high μ is usually achieved by using currently the most popular, usually the highest yielding, cultivars as parent(s), while a high level of σ_*G*_ is achieved by choosing parents that are different and complementary in yield components, agronomic traits, disease resistances, and quality traits, and by use of a large enough breeding population. Crossing an adapted local cultivar with a geographically distant cultivar with desired traits led to some of the most important wheat cultivars in China (Zhao et al., 1981). In the era of genomic selection, μ and σ_*G*_ and therefore *P* can be predicted for any pair or set of potential parents for a trait of interest if reliable genomic models are available (Wang et al., 2018).

Usually, the genetic variability in a breeding population is created by crossing different parents, but it can also be created through induced mutations by treating a superior cultivar with γ radiation, chemical mutagen treatment, transposons, genetic transformation, or gene editing (e.g., van Harten, 1998; Kharkwal et al., 2004; Shu et al., 2012).

To maximize μσ_*G*_ may suggest that μ and σ_*G*_ are equally important. In cultivar development, however, μ may be more important than σ_*G*_ although both are essential. The use of backcross, recurrent selection, and crosses between closely related breeding lines (e.g., Rasmusson and Phillips, 1997) are examples to ensure a high μ at the expense of σ_*G*_. On the contrary, wide crosses (e.g., Baum et al., 1992) can bring much variability to the population at the expense of reduced population mean. Wide crosses are essential to introduce novel genes and traits from wild species (e.g., Ma et al., 2018; numerous research done worldwide for various crops), which are crucial to long-term crop improvement; however, they are unlikely to directly lead to superior cultivars.

## Selection Gain, Selection Efficiency, Selection Intensity, Culling Rate, and Heritability

Equation 2 or the second part Equation 1 consists of factors determining the selection gain and is known as the Breeder's Equation. It may be more accurately referred as the breeder's equation for progeny selection. Here σ_*G*_ is fixed for a given breeding population, *i* is a parameter artificially set, and *h* is the square root of achieved heritability. In fact, while *i* is the artificially set selection intensity, *ih* is the realized selection intensity. Cobb et al. (2019) discussed approaches to improving breeding efficiency in the framework of Equation 2, with the emphasis on reducing *Y*. Rutkoski (2019) reviewed the basis and approaches to achieve genetic gain.

**Figure 1:**
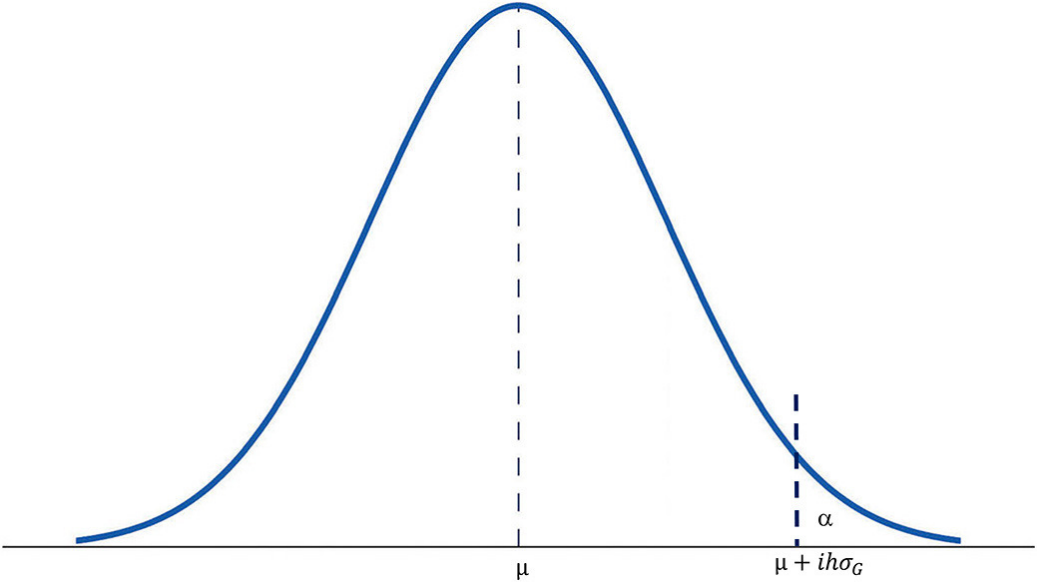
A chart of normal distribution to show the relationships among various parameters in the Complete Breeder's Equation. μ is the mean of the breeding population; σ_*G*_ is the square root of the genotypic variance of the population; *i* is the artificially set selection intensity in the unit of σ_*G*_; *h* is the square root of achieved heritability (*h*^2^ or *H*); α is the portion of the population to be selected; it is also the probability that the best genotypes are not included in the selected portion.

Equation 1 can be better understood from Figure 1. Assume that the breeding population is normally distributed with a mean μ and a variability σ_*G*_. The X-axis is the range of the phenotypic values and the Y-axis is the frequency density. The area under the curve is unity (1 or 100%). With a selection intensity *ih*, genotypes to be culled lie on the left side of the vertical line defined by *x* = μ + *ihσ*_*G*_, and genotypes to be retained lie on the right side of the line. The area α is the proportion of the population to be retained and 1 − α is proportion to be culled. α is also the probability for *ihσ*_*G*_ < 0, while 1 − α is the probability for *ihσ*_*G*_ > 0. In other words, α is the probability for a genotype with a phenotypic value of μ + *ihσ*_*G*_ to be no better than the population mean.

An extended interpretation is that α is the risk that the best genotype in the population is *not* retained at the selection intensity *ih*. Apparently, the risk is reduced as *ih* is increased while *h* is the only objective variable. If *h* = 0, then *ih* = 0, and α = 50%. As *h approaches unity*, α approaches 0. This provides a clue for the choice of *i*. According to the normal distribution table, if α *is set to* 0.0001, then *z* = *ih* = 3.7. Therefore, it is rational to set *i* = 3.7 at α = 0.0001. The relationships between heritability, *ih*, and α at *i* = 3.7 for some selected heritability values are listed in Table 1, assuming a population size of *n* = 10, 000.

**Table 1:** The realized selection intensity (*z* = *ih*), the proportion of the population to be retained (α), and the number of genotypes to be retained (*N*) at different levels of heritability (*H or h*^2^) assuming a population of *n* = 10, 000 and a selection intensity of i=3.7.

***H* = *h*^2^**	**h**	***z* = 3.7*h***	**(1-α)**	**α (%)**	**N (n = 10,000)**	**Corresponding breeding stages in Yan et al. (2019b)**
0.0	0.00	0.00	0.5000	50.00	5000	
0.1	0.32	1.17	0.8790	12.10	1210	Stage 3.1 (yr1)
0.2	0.45	1.65	0.9505	4.95	495	
0.3	0.55	2.03	0.9788	2.12	212	Stage 3.2 (yr2)
0.4	0.63	2.34	0.9904	0.96	96	
0.5	0.71	2.62	0.9959	0.41	41	Stage 4.1 (yr3)
0.6	0.77	2.87	0.9980	0.21	21	Stage 4.2 (yr4)
0.7	0.84	3.10	0.9990	0.10	10	State 4.3 (yr5)
0.8	0.89	3.31	0.9995	0.05	5	Stage 4.4 (yr6)
0.9	0.95	3.51	0.9998	0.02	2	Stage 4.5 (yr7)
1.0	1.00	3.70	0.9999	0.01	1	Cultivar release

If α is interpreted as the percentage of the population that must be retained to ensure that the best genotype(s) is included, then the number of genotypes must be selected, *N*, will be:

(4) $$\begin{array}{l} N=n\alpha , \end{array}$$

where *n* is the effective population size, i.e., the number of unique genotypes in the breeding population. The inverse of *N* may be defined as the rate of selection success (Yan et al., 2019b):

(5) $$\begin{array}{l} S\;=\;1/N. \end{array}$$

For example, for *n* = 10, 000, *H* = 0.9, and *i* = 3.7, we have α = 0.02% and N = 2 (Table 1). That is, for a population of 10,000 unique genotypes, an achieved heritability of 0.9 would guarantee that the best genotype is between the top two. A smaller *N* means less time (in years) and cost that are needed to single out the best genotype. In the extreme case, if a selection method (genomic prediction or any other approach) is accurate enough to identify the best genotype (i.e., *N* = 1) out of a breeding population, then all the time and cost associated with subsequent testing would be saved. In contrast, in the Ottawa oat breeding program, it takes about seven years of visual selection and yield trials to identify the best genotypes out of a breeding population (Yan et al., 2019b; last column of Table 1, this paper). Each year ~10,000 F_2_ derived breeding lines are planted in a hill nursery and 1,000 are visually selected in the field and the seed lab. Assuming that the best genotype is included in these selected lines, the rate of selection success for this stage (the “Hill Nursery” stage or Stage 3.1) is ~1/1000, roughly corresponding to assuming an *H* = 0.1 (Table 1). The 1,000 selected lines are then planted in yield plots and ~200 lines are visually selected (the “Observation Plot” stage or Stage 3.2). The accumulative rate of selection success for these two years of visual selection is, therefore, ~1/200, corresponding to *H* = 0.3 (Table 1). It takes four to five additional years of multi-location test to single out the best few genotypes as potential new cultivars (Stages 4.1 to 4.5 in Table 1). Experience indicates that the top genotypes at the Stage 4.3 are usually the ones to be released as cultivars; this corresponds to *H* = 0.7 (Table 1). Trials in Stages 4.4 and 4.5 (years 2 and 3 of the Registration Test) are conducted mainly to verify the results and to obtain data required for official variety registration.

Genomic selection applied at the Hill Nursery stage (Stage 3.1) is expected to dramatically improve the rate of selection success so as to reduce the number of years spent in visual selection and yield trials (*Y*, Equations 1 and 2). A minimum requirement for a viable genomic selection procedure is to improve the selection efficiency to an extent that covers the extra cost spent in genotyping, phenotyping, and model development. Alternatively, genomic selection is justified if it can identify the best genotypes that may be discarded by the breeder's eye.

**Figure 2:**
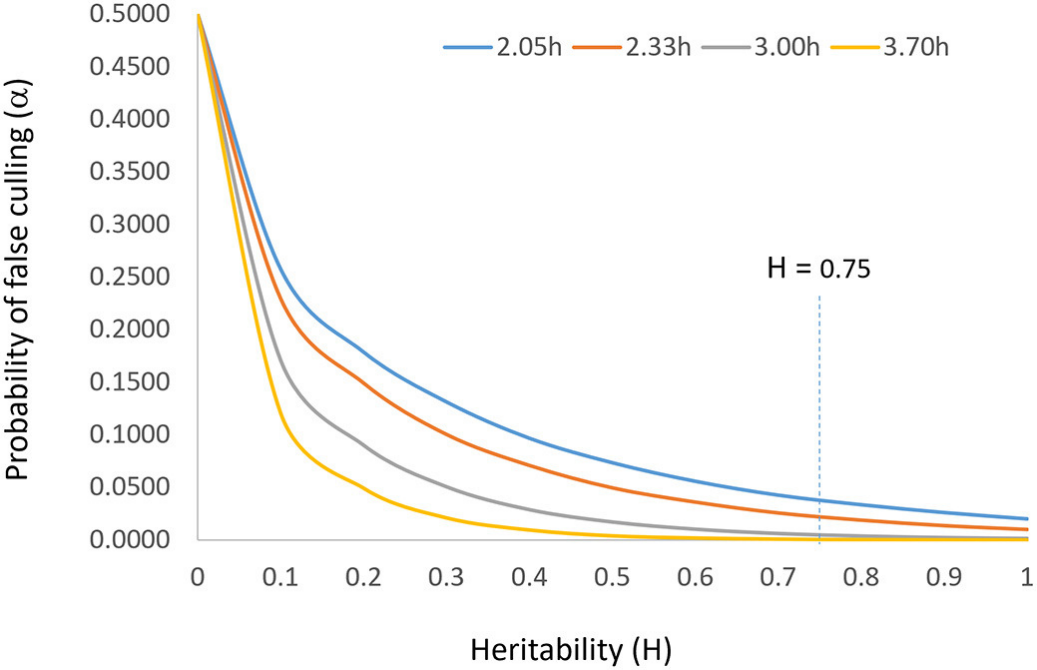
The relationship between heritability (H or *h*^2^) and probability of false culling (α) at four levels of selection intensity (*i*). It is suggested that the probability of false culling that can be tolerated be set according to the population size (*n*) such that α = 1/*n*; *i* can then be determined by α by consulting the normal distribution table. The vertical line of H = 0.75 indicates the target heritability to be achieved for reliable selection.

The parameter *i* should be set according to the population size such that α = 1/*n*. This reflects the idea that a larger population allows a higher selection intensity at the same level of heritability and that the top genotype is the best genotype (*N* = 1) when *h* = 1. According to the normal distribution table, *i* should be set at 2.05, 2.33, 3.00, and 3.71 when *n* = 50, 100, 1,000, and 10,000, respectively. The relationship among heritability, selection intensity, and probability of false culling (α) is displayed in Figure 2. Incidentally, Singh and Chaudhary (1977) suggested setting *i* = 2.063 at α = 0.05, in line with this idea.

Alternatively, the achieved heritability may be used as the culling rate when the population is small; the number of genotypes that must be retained can then be roughly estimated by:

(6) $$\begin{array}{l} N=n\left(1-h^{2}\right). \end{array}$$

When *h*^2^ = 0, no genotype would be discarded because the selection is completely unreliable; and when *h*^2^ = 1, all but the top performing genotype can be discarded because any observed difference is genetic and heritable. For example, if *n* = 40 and *h*^2^ = 0.95, then 95% or 38 of the 40 entries can be discarded and the top two performing genotypes can be selected or recommended.

To summarize, for a given breeding population and a given target environment, the allowable culling rate, the allowable selection intensity, the achievable rate of selection success, and the expected selection gain are all determined solely by the achieved heritability, in a curvilinear fashion (Figure 2). Therefore, heritability is the single most important concept in progeny selection.

## Heritability Under the Multi-Location, Multi-Year Framework

Cultivars are developed to adapt to a specific target region, i.e., to the environments that may be encountered across locations and years in a target region. Therefore, the heritability discussed so far must be defined in the multi-location, multi-year framework (Comstock and Moll, 1963; DeLacy et al., 1996; Atlin et al., 2000). According to the general linear model, a phenotype, i.e., an observed value, is a mixed effect of environmental main effect (E), genotypic main effect (G), genotype by environment interaction (GE), and experimental error (ε), where E is the sum of location main effect (L), year main effect (Y), and their interaction (LY). Assuming orthogonal experimental design i.e., the same set of genotypes are tested at the same set of locations each year with the same number of replicates, the phenotypic variance is $\sigma_{P}^{2}=\sigma_{G}^{2}+\frac{\sigma_{GL}^{2}}{l}+\frac{\sigma_{GY}^{2}}{y}+\frac{\sigma_{GLY}^{2}}{ly}+\frac{\sigma_{\varepsilon }^{2}}{lyr}$. The entry-mean heritability, i.e., the proportion of phenotypic variance that can be explained by the genetic variance at the entry mean level, is estimated by (Fehr, 1991; DeLacy et al., 1996):

(7) $$\begin{array}{l} H_{rly}=\frac{\sigma_{G}^{2}}{\sigma_{P}^{2}}=\frac{\sigma_{G}^{2}}{\sigma_{G}^{2}+\frac{\sigma_{GL}^{2}}{l}+\frac{\sigma_{GY}^{2}}{y}+\frac{\sigma_{GLY}^{2}}{ly}+\frac{\sigma_{\varepsilon }^{2}}{rly}}, \end{array}$$

where *H*_*rly*_ stands for heritability across *l* locations in *y* years with *r* replicates; $\sigma_{GL}^{2},\;\sigma_{GY}^{2},\;\text{and}\;\sigma_{GLY}^{2}$ are the variances for the genotype by location interaction (GL), genotype by year interaction (GY), and genotype by location by year interaction (GLY), respectively; and $\sigma_{\varepsilon }^{2}$ is the variance for experimental error.

When trials are not conducted orthogonally regarding genotypes, location, years, or replicates, which is usually the case, each trial (location-year combination) may be considered as an environment, and the heritability can be estimated by

(8) $$\begin{array}{l} H_{rly}=\frac{\sigma_{G}^{2}}{\sigma_{G}^{2}+\frac{\sigma_{GE}^{2}}{\sum_{i=1}^{y}l_{i}}+\frac{\sigma_{\varepsilon }^{2}}{\sum_{i=1}^{y}\sum_{j=1}^{l_{i}}r_{ij}}}, \end{array}$$

where $\sigma_{GE}^{2}$ is the variance for genotype by environment interaction. For convenience, Equation 7 will be used in further discussions. Restricted maximum likelihood (REML) is the preferred method for estimating the various variances, particularly when the data are unbalanced (e.g., Gilmour et al., 1995). REML is implemented in all software packages with a mixed model procedure.

Heritability for a single trial can be estimated by

(9) $$\begin{array}{l} H_{r}=\frac{\sigma_{G}^{2}}{\sigma_{G}^{2}+\frac{\sigma_{\varepsilon }^{2}}{r}}, \end{array}$$

However, *H*_*r*_ can be used to assess the data quality of a trial but not for making final selections. For making section decisions, Equation 7a below should be used instead:


$$\begin{array}{l} H_{rly}=\frac{\sigma_{G}^{2}}{\sigma_{G}^{2}+\frac{\sigma_{GL}^{2}}{1}+\frac{\sigma_{GY}^{2}}{1}+\frac{\sigma_{GLY}^{2}}{1}+\frac{\sigma_{\varepsilon }^{2}}{r}}.\;\left[7a\right] \end{array}$$

That is, although the interaction terms cannot be estimated from a single trial, they must be factored in when making selection decisions. It can be seen that *H*_*r*_ is an inflated estimation of *H*_*rly*_ for a trial because the denominator in Equation 7a should be much larger than that in Equation 9.

Likewise, a heritability can be estimated for multi-location trials conducted in a year,

(10) $$\begin{array}{l} H_{rl}=\frac{\sigma_{G}^{2}}{\sigma_{G}^{2}+\frac{\sigma_{GL}^{2}}{l}+\frac{\sigma_{\varepsilon }^{2}}{rl}}, \end{array}$$

but it is not to be used to make final selection decisions. Instead, equation 7b should be used,


$$\begin{array}{l} H_{rly}=\frac{\sigma_{G}^{2}}{\sigma_{G}^{2}+\frac{\sigma_{GL}^{2}}{l}+\frac{\sigma_{GY}^{2}}{1}+\frac{\sigma_{GLY}^{2}}{l}+\frac{\sigma_{\varepsilon }^{2}}{rl}}.\;\left[7b\right] \end{array}$$

*H*_*rl*_ is an inflated estimation of *H*_*rly*_ for a single-year test. The definition of heritability in the form of Equation 7 is the only valid definition to be used in Equation 1; Figure 1, and Table 1, with $h=\sqrt{H_{rly}},\;$ even though *H*_*rly*_ cannot be directly estimated in some stages of the breeding cycle. It should be noted that the definition of heritability is in line with the concept of mixed models. It consists of variances for G, GE (= GL + GY + GLY), and experimental error but excludes that for E (= L + Y + LY), implying a mixed model. It implies that G, GE, and experimental error are considered as random effects but E as fixed effects (DeLacy et al., 1996). Researchers are often puzzled on which effects should be treated as random and which fixed when analyzing multi-environment trials data using mixed models (Piepho et al., 2003); for the purpose of genotype evaluation, this is clear from the definition of heritability. The definition of heritability is also consistent with the concept of GGE biplot analysis, which excludes E and focuses on G and GE for cultivar and test environment evaluation (Yan et al., 2000; Yan and Kang, 2002; Yan and Tinker, 2006; Yan, 2014).

All efforts made to improve selection efficiency are also efforts to improve the heritability as defined in Equation 7 or Equation 8. Put it differently, all possible approaches to improve selection efficiency reside in the definition of heritability. These include approaches to deal with GE and approaches to minimize experimental error. Dealing with GE include two steps: (1) identifying and utilizing repeatable GE, a process often referred to as mega-environment analysis (Yan, 2014, 2015, 2016, 2019), and (2) accommodating unrepeatable GE through adequate testing (Yan et al., 2015; Yan, 2016, 2021). Dealing with experimental error includes adequate replication (Yan et al., 2015; Yan, 2021) and spatial variation adjustment (Cullis and Gleeson, 1991; Gilmour et al., 1997; Cullis et al., 1998; Burgueño et al., 2000; Qiao et al., 2000; Yang et al., 2004; Yan, 2014).

## Mega-Environment Analysis and Utilization of Repeatable GE

### Repeatable GE vs. Unrepeatable GE

Mega-environment analysis is analysis of the G+GE patterns aiming at dividing a target region into meaningful subregions or mega-environments (subregions and mega-environments are used interchangeably in this article). Among the components of GE, GY and GLY are obviously unrepeatable because it is impossible to predict the environments of next year. It is possible, though, that some of the GL is repeatable as the soil and daylength at a location are fixed. Some management factors such as irrigation, fertilizer application, and fungicide application may also lead to repeatable GE (Cooper et al., 2021), which are lumped as “common management practices in the target region or mage-environment” for simplicity. Assuming that the test locations can be divided into two or more groups (subregions), the variance for GL will be divided into variance for genotype by subregion interaction ($\sigma_{GS}^{2}$), which is the repeatable part, and genotype by location interaction within subregions ($\sigma_{GL\left(s\right)}^{2}$), which is the unrepeatable part, of GL (Atlin et al., 2000; Yan, 2016):

(11) $$\begin{array}{l} \sigma_{GL}^{2}=\sigma_{GS}^{2}+\sigma_{GL\left(s\right)}^{2} \end{array}$$

and the number of test locations *l* will also be divided among the subregions:

(12) $$\begin{array}{l} l=\overset{s}{\underset{\text{k}=1}{\sum }}l_{k} \end{array}$$

where *l*_*k*_ is the number of test locations within subregion *k*. Subdivision of the target region into subregions will improve the overall heritability if the genotype by subregion interaction is sufficiently large, because the genotype by subregion interaction is converted into genotypic main effect within subregions when genotype evaluation is conducted by subregion:

(13) $$\begin{array}{l} H_{rly}^{{}^{\prime }}=\frac{\sigma_{G}^{2}+\sigma_{GS}^{2}}{\left(\sigma_{G}^{2}+\sigma_{GS}^{2}\right)+\frac{\sigma_{GL\left(S\right)}^{2}}{l}+\frac{\sigma_{GY}^{2}}{y}+\frac{\sigma_{GLY}^{2}}{ly}+\frac{\;\sigma {\;}_{\varepsilon }^{2}}{rly}} \end{array}$$

where $H_{rly}^{{}^{\prime }}$ is the entry-mean heritability when genotype evaluation is conducted by subregion. On the other hand, dividing a region into subregions may lead to reduced heritability within a subregion due to the smaller number of test locations (Equation 12). Thus, Atlin et al. (2000, 2011) warned that subdivision of a target region should be avoided if genotype by subregion interaction is small relative to G. They used the genetic correlation between divided subregions and the undivided whole region (*r*_*G*_) as a measure to decide whether the target region should be divided, which is defined as:

(14) $$\begin{array}{l} r_{G}=\sqrt{\frac{\sigma_{G}^{2}}{\sigma_{G}^{2}+\sigma_{GS}^{2}}} \end{array}$$

They suggested that subdivision should be avoided if *r*_*G*_ is high, although an explicit criterion was not given. In fact, the correlation between candidate subregions should be a more meaningful measure.

Nevertheless, if a subregion is found to be distinct from other subregions, it should be treated as such; if a subregion is economically important, it is justifiable to increase the number of test locations within it to achieve a sufficiently high heritability or selection reliability. The merit of dividing a target region into meaningful subregions is to allow selection and deployment of subregion-specific cultivars to achieve a higher genetic gain within each subregion and thereby the whole region. Annicchiarico (2021) presented a recent example that selection for mega-environment specific cultivars increased genetic gains, in addition to a good review on the subject matter. An essential condition for dividing a target region into subregions is the presence of substantial crossover genotype by subregion interactions (discussed below).

### How to Reveal Repeatable GE

To investigate whether heritability can be improved by dividing a target region into subregions, the prerequisite is a good hypothesis on how to divide the target region. Various approaches have been used in dividing a jurisdictional region into agroclimatic regions as reviewed in Yan et al. (2011). A poor hypothesis will lead to the false conclusion that the target region cannot be divided and thereby miss the opportunity to utilize the repeatable GE. For example, Atlin et al. (2000) hypothesized that western Canada (including Alberta, Saskatchewan, and Manitoba) and eastern Canada (including Ontario, Quebec, and Maritime provinces) were two barley mega-environments and rejected the hypothesis. Based on the same dataset, however, Yan and Tinker (2005) showed two clear mega-environments, with locations in Alberta and Saskatchewan as one mega-environment and locations in Manitoba and the eastern Canadian provinces as the other. For another example, in analyzing the data of a set of maize hybrids tested at 24 sites in six African countries in 2009, Atlin et al. (2011) hypothesized that each country was a mega-environment and concluded that there was no mega-environment differentiation. However, a country is a political entity rather than an ecoclimatic region, so the hypothesis *per se* is questionable. A good hypothesis on mega-environment differentiation must be based on the G+GE patterns. Two methods have been developed to reveal repeatable GE patterns: GGE + GGL biplot analysis (Yan, 2014, 2015, 2016) and LG (location-grouping) biplot analysis (Yan, 2019; Yan et al., 2019b, 2021).

#### GGE + GGL Biplot Analysis

As the definition of heritability in Equation 7 or Equation 8 suggested, data from multi-location, multi-year trials are required to conduct GGE + GGL biplot analysis, where GGE stands for G + GE (meaning fitting G + GE by principal components), and GGL for G + GL. In variety trials, usually the same set of genotypes are tested at all locations in a year but different sets of genotypes are tested in different years, because poor genotypes are dropped and new genotypes added each year. Consequently, multi-location, multi-year data are typically unbalanced. Nevertheless, usually a sizable number of common genotypes are tested in two or more consecutive years; this allows missing values in the genotype by environment two-way table to be imputed based on certain procedures (e.g., Yan, 2013). Data from such trials can be investigated using a GGE + GGL biplot (Yan, 2014, 2015, 2016), as shown in the example below.

**Figure 3:**
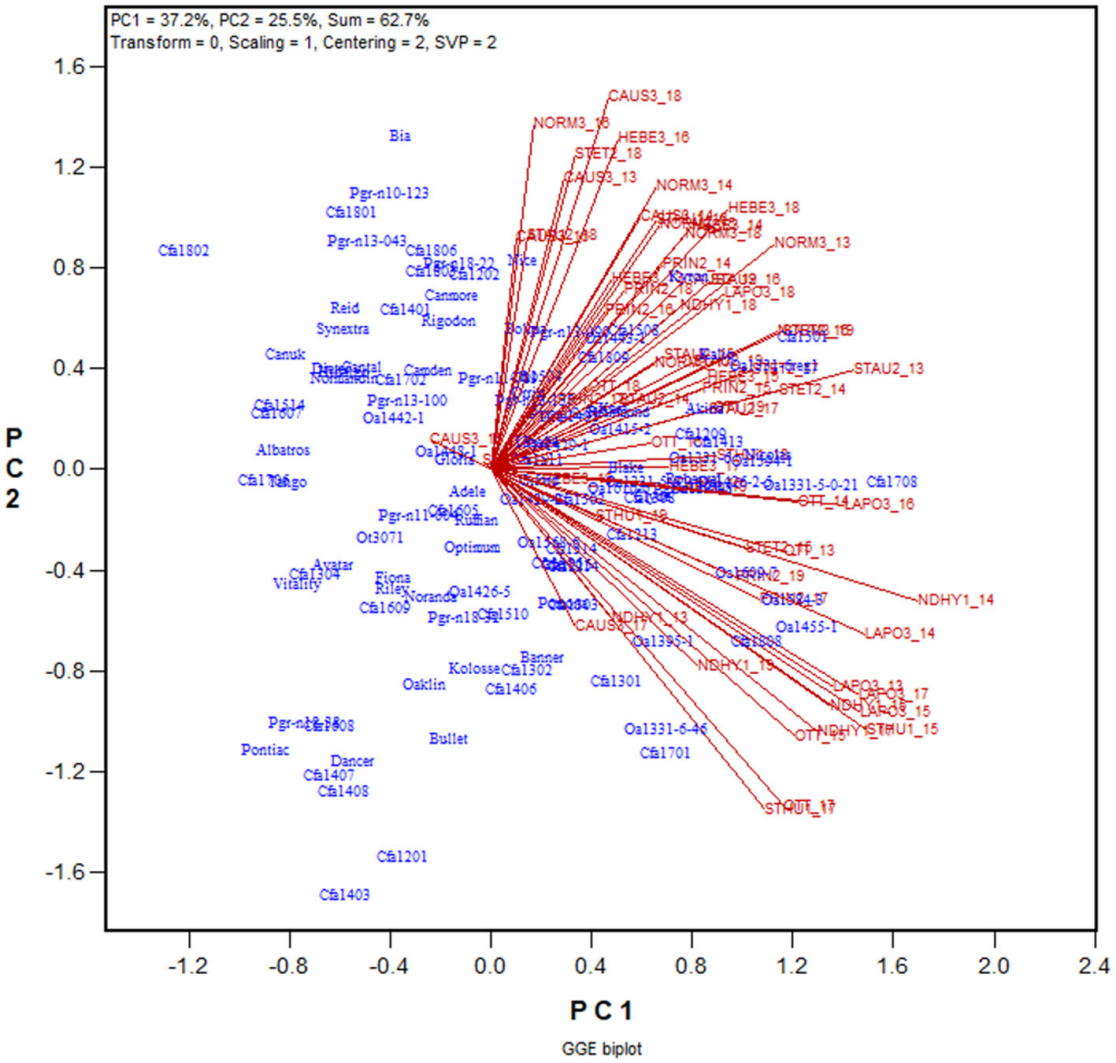
GGE biplot to show the relative yield of 116 oat genotypes in 67 trials from the 2013–2019 Quebec provincial oat trials. The genotypes are displayed in blue and the trials in red. Each trial is displayed as a location-year combination. The Quebec locations are: NDHY1 (St Hyacinthus) and STHU1 (St. Huber) in Zone 1, PINT2 (Pintendre), PRIN2 (Princeville), STAU2 (St. Augusta), and STET2 (St. Etienne) in Zone 2, and NORM3 (Normandin), CAUS3 (Causapscal), HEBE3 (Hebertville), and LAPO3 (La Pocatière) in Zone 3. OTT (Ottawa) is a location in Ontario. PC1 and PC2 are the first two principal components from singular value decomposition of the trial-standardized yield data (“Scaling = 1,” “Centering = 2”), with the singular values fully partitioned to the trial scores (“SVP = 2”) for proper visualization of the correlations among trials.

**Figure 4:**
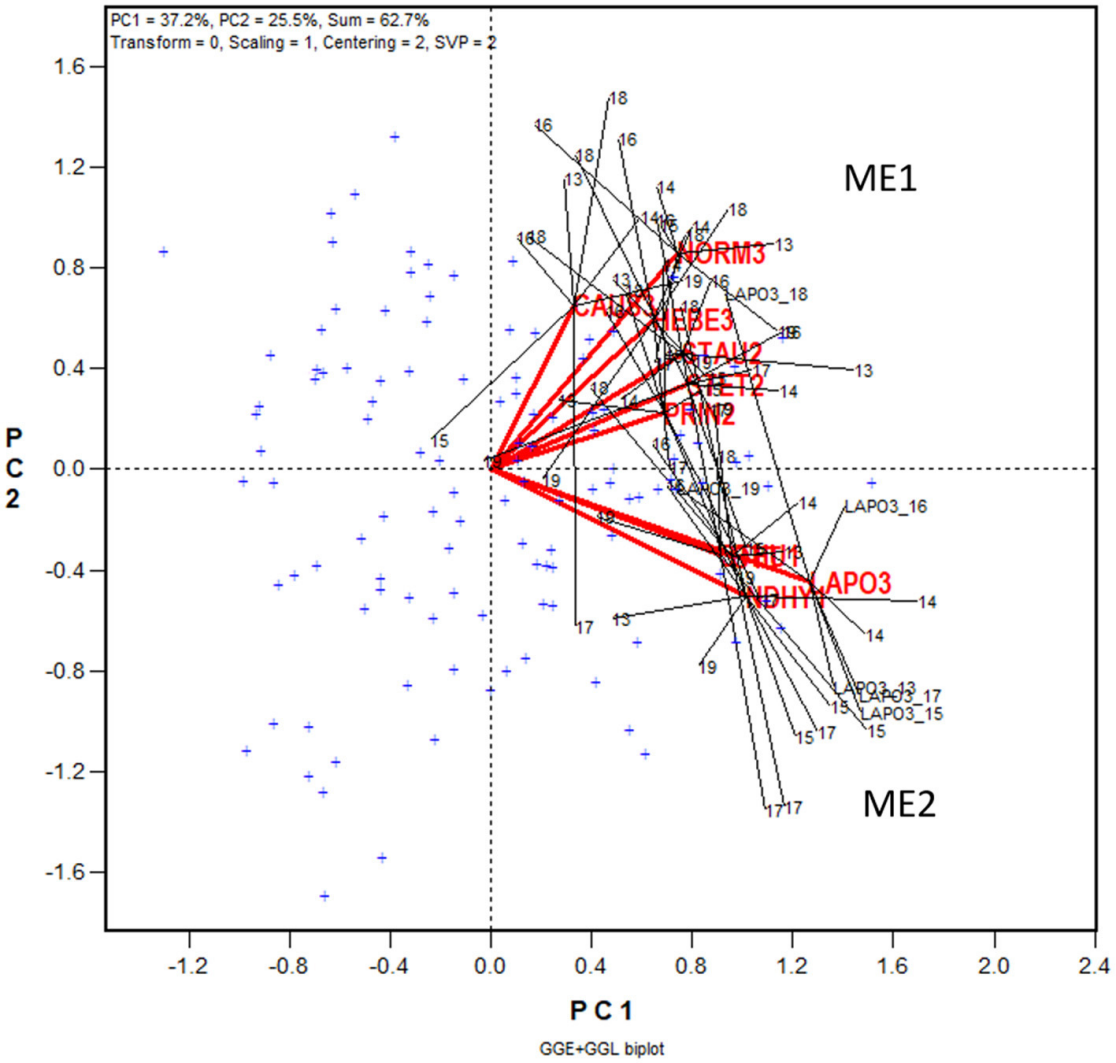
GGE + GGL biplot modified from Figure 3 to show two groups of locations or oat mega-environments (ME) in Quebec. Mega-environment 1 (ME1) consists of Zone 2 and Zone 3 locations PINT2, PRIN2, STAU2, STET2, CAUS3, HEBE3, and NORM3, and mega-environment 2 (ME2) includes locations NDHY1, STHU1, LAPO3, and OTT. The trials conducted at each location are presented as a cluster of trials, with the location name placed at the center and the individual trials, indicated by the last two digits of the year, placed around it, and the trials are connected to the center with straight lines. Two locations are regarded as belonging to the same mega-environment if their clusters overlap; they are regarded as belonging to different mega-environments otherwise. The variation in the placement of the locations between mega-environments represents repeatable GE and the variation in the placement of the trials within mega-environments represents unrepeatable GE. The genotypes are displayed as “+” in blue for clarity.

The yield data from the 2013 to 2019 Quebec Oat Registration and Recommendation trials are used here as an example (data available from the author upon request). Each year 41 to 46 registered oat cultivars or breeding lines were tested at eight to 10 locations. The locations represent three ecoclimatic zones of Quebec (Yan et al., 2011; Yan, 2015). Zone 1 was represented by NDHY1 (St Hyacinthus) and STHU1 (St. Huber), Zone 2 by PRIN2 (Princeville), PINT2 (Pintendre), STAU2 (St. Augusta), and STET2 (St. Etienne), and Zone 3 by NORM3 (Normandin), HEBE3 (Hebertville), CAUS3 (Causapscal), and LAPO3 (La Pocatière); the number at the end of each location code indicates the zone it belongs. In addition, the trials were also conducted at OTT (Ottawa in Ontario), which is geographically close to Zone 1 of Quebec. A total of 118 genotypes and 67 trials (location by year combinations) were involved in these seven years, forming a 118-genotype by 67-trial two-way table, with 63% missing values. The first step of the analysis was to generate a GGE biplot containing the 118 genotypes and the 67 trials (Figure 3). The GGE biplot was constructed by the first two principal components from subjecting the trial-standardized genotype by trial two-way table to singular value decomposition, after proper singular value partition (Yan, 2002). The most obvious message from this fairly crowed biplot is that the trials placed on the upper portion of the biplot and those on the lower portion were negatively correlated, as indicated by the obtuse angles between them. This indicates existence of strong GE. The second step is to summarize the trials conducted at a location by a location marker, the placement of which is determined by the mean coordination of the trials (Figure 4). For example, the placement of the location LAPO3 (in red) was determined by the seven trials conducted at LAPO3, namely LAPO3_13, LAPO3_14, LAPO3_15, LAPO3_16, LAPO3_17, LAPO3_18, and LAPO3_19 (in black). The genotypes are represented by “+” for clarity. The biplot in Figure 4 is both a GGE biplot and a GGL biplot, thus the term GGE+GGL biplot.

In Figure 4 the 10 locations are clearly separated into two groups: group 1 include locations NORM3, HEBE3, CAUS3, PRIN2, PINT2, and STAU2 on the upper quadrant, and group 2 include locations NDHY1, STHU1, LAPO3, and OTT on the lower quadrant. Thereby the GE is divided into repeatable GE and unrepeatable GE. The genotype by location group interaction, i.e., the difference in the placement between the two location groups, is the repeatable GE; the genotype by trial interaction within groups, i.e., the variation in the placement among the trials within each of the two location groups, is the unrepeatable GE. The two location groups suggests two different mega-environments. All locations in mega-environment 1 (ME1) belong to Zone 2 or Zone 3 of Quebec; locations in mega-environment 2 (ME2) consists of two Zone 1 locations, a Zone 3 location, and OTT. Thus, the mega-environment differentiation was largely, but not completely, consistent with the agroclimatic zones.

#### LG Biplot Analysis

**Figure 5:**
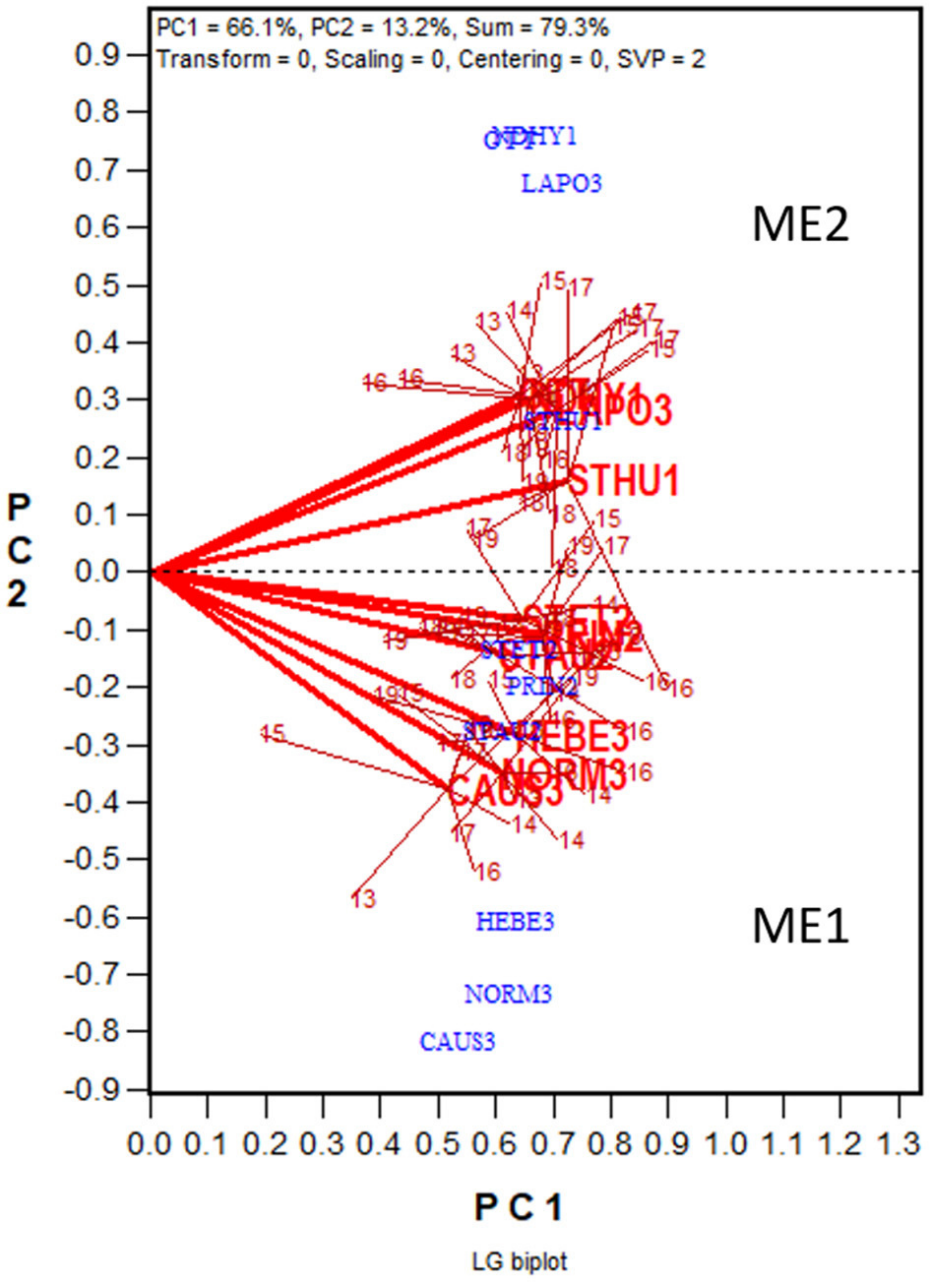
LG biplot to show two oat mega-environments in Quebec. PC1 and PC2 are the first two principal components from singular value decomposition of the location by trial two-way table of correlations, without centering (“Centering = 0”) or scaling (“Scaling = 0”). The LG biplot is a location by trial biplot, with the locations presented in blue and the trials in red. The trials conducted at each location are presented as a cluster of trials, with the location name placed at the center and the individual trials, indicated by the last two digits of the year, placed around it. The trials are connected to the location with straight lines. Two locations are regarded as belonging to the same mega-environment (ME) if their clusters overlap; they are regarded as belonging to different mega-environments otherwise. The same two MEs (ME1 and ME2) shown in Figure 4 are shown in this LG biplot. The variation in the placement of the locations between mega-environments represents repeatable GE and the variation in the placement of the trials within mega-environments represents unrepeatable GE.

Presented in Figure 5 is the LG biplot based on the same dataset that was used to generate the GGE + GGL biplot (Figure 4). The steps to construct the LG biplot follows. First, a genetic correlation matrix among locations was calculated for each year. Second, the yearly correlation matrices were stacked to form a location by trial two-way table of correlations, each trial being a location-year combination. Third, the location by trial table was submitted to singular value decomposition, without entering or scaling (“centering = 0, scaling = 0”). Fourth, the resulting first two principal components were used to construct a location by trial biplot. Fifth, as in the GGE + GGL biplot, the trials conducted at a location in different years were summarized by a location marker defined by the mean coordination of the trials. Finally, the trials at a location are displayed as a cluster, with the location marker as the center and the trials in different years as members; the trials and the location are connected with straight lines. If the clusters of two locations overlap, they are regarded as belonging to the same mega-environment; if they do not overlap, they are considered as belonging to different mega-environments. In the LG biplot, the variation among trials and locations within a mega-environment, i.e., the variation among trials at overlapping locations, represents unrepeatable GE; the variation between two mega-environments represents repeatable GE. It can be seen that the same two mega-environments shown in the GGE+GGL biplot (Figure 4) are clearly separated in the LG biplot (Figure 5); thus, the two approaches are functionally equivalent or similar. Importantly, the LG biplot has the advantage that it does not require any common genotypes to be tested in different years. Therefore, it can be used to reveal repeatable GE and delineating mega-environments using multi-year trial data in which completely different sets of genotypes are tested in different years (Yan et al., 2021).

A general comment on the use of biplots follows. A 2-D biplot is usually used for data visualization for convenience and on the understanding that the most important patterns in the data are captured by the first two principal components. However, there may be cases where some important patterns exist in higher order principal components. This is usually indicated by the presence of vectors that are obviously shorter than others. When this is the case, variation not displayed in the biplot can be explored by biplots displaying a subset of the data. A recent example can be found in Yan et al. (2021).

### Utilization of Repeatable GE by Selecting Mega-Environment Specific Cultivars

**Figure 6:**
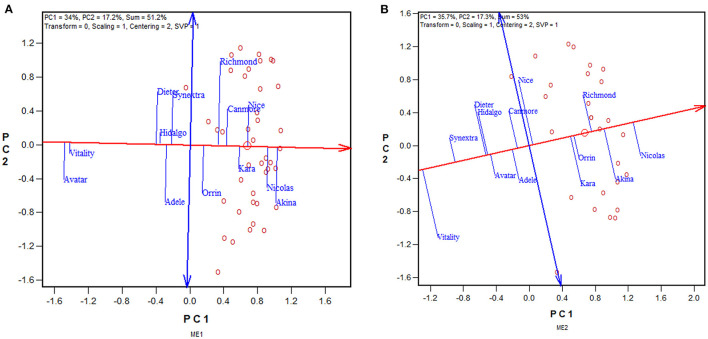
GGE biplots to show the mean yield and instability of 13 oat cultivars in **(A)** mega-environment 1 (ME1) and **(B)** mega-environment 2 (ME2), across 2013–2019. See Figure 4 and associated text for the definitions of ME1 and ME2. PC1 and PC2 are the first two principal components from singular value decomposition of trial-standardized yield data (“Centering = 2,” “Scaling = 1”). The singular values were entirely partitioned to the genotypic scores (“SVP = 1”) for proper genotype evaluation. The trials are represented by “o” for clarity. The red line with a single arrow is the average environment axis (AEA), the arrow pointing to higher mean yield. The blue line with arrows on both ends is the instability axis, the arrows pointing to greater instability in either direction.

The approach to utilizing repeatable GE is to select separately for each mega-environment, preferably using the mean vs. stability view of the GGE biplot (Figure 6). The red line with a single arrow passes through the biplot origin and the mean environment (which has mean coordination of all environments) and is referred to as the average environment axis (AEA) or GGE-Mean axis; the arrow points to higher mean yield. The blue line with arrows at both ends points to greater instability in either direction; it can be referred as the GGE-stability axis (Yan, 2001; Yan and Kang, 2002; Yan and Tinker, 2006). This is an extended application of the inner-product property of a biplot (Gabriel, 1971). Thus, the three highest yielding cultivars in ME1 across 2013–2019 were Akina > Nicolas > Nice (Figure 6A), and those for ME2 were Nicolas > Akina > Richmond (Figure 6B). Therefore, the repeatable GE can be utilized by recommending different sets of cultivars in ME1 and ME2.

**Figure 7:**
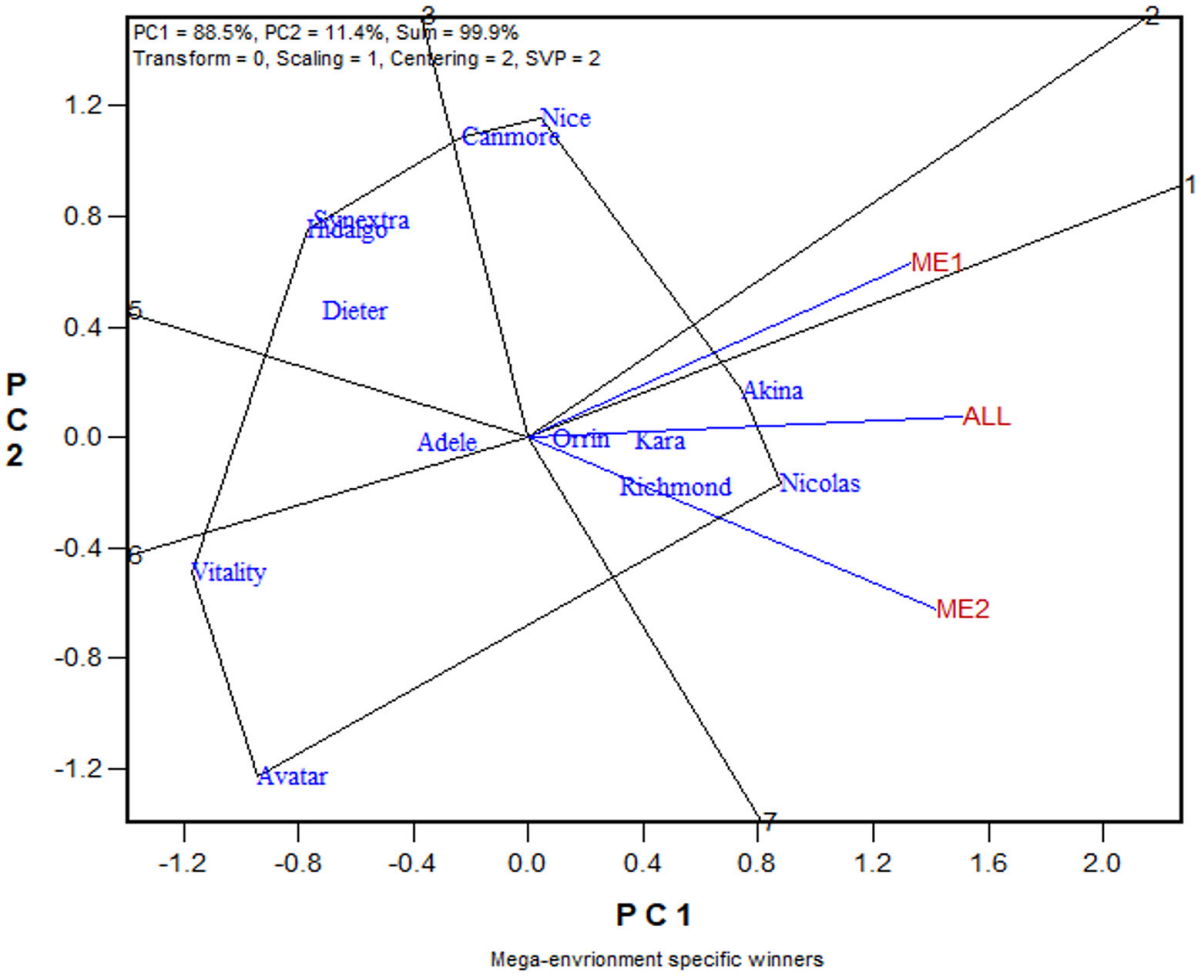
The which-won-where view of the GGE biplot to show the relative yield of 13 oat cultivars in mega-environment 1 (ME1), mega-environment 2 (ME2), and the undivided Quebec oat growing regions (ALL). The polygon was formed by connecting the genotypes that are placed away from the biplot origin in all directions. For each polygon side a line perpendicular to it was drawn from the biplot origin. These lines dissect the biplot into sectors. For each sector, the genotype at the vertex is the nominal highest yielder for the environments or mega-environments fell in it. In this case, Akina was the highest yielder in ME1 while Nicolas was the highest yielder in both ME2 and “ALL.”

The similarity/dissimilarity in cultivar ranking between ME1 and ME2, along with that in the undivided whole region, are further presented in the which-won-where view of the GGE biplot in Figure 7. The polygon or which-won-where view of the GGE biplot (Yan et al., 2000) is also an extended application of the inner-product property of a biplot (Gabriel, 1971). The polygon was formed by connecting the genotypes that are placed away from the biplot origin in all directions. For each polygon side a line perpendicular to it was drawn from the biplot origin. These lines dissect the biplot into sectors. For each sector, the genotype at the vertex is the nominal highest yielder for the environments or mega-environments fell in it. In this case, Akina was the highest yielder in ME1 while Nicolas was the highest yielder in ME2 and “ALL,” indicating crossover genotype by subregion interaction. On the other hand, ME1 was placed close to the radiate line labeled “1,” which separates ME1 from ME2; this means that Akina had higher yield than Nicolas in ME1 but not by much. The two mega-environments were moderately correlated (*r* = 0.652; Figure 7) and shared Akina and Nicolas as the top two yielding cultivars, though in a reversed order. Thus, the two oat mega-environments in Quebec were classified as sub mega-environments within one of the three major oat mega-environments in Canada (Yan et al., 2021).

## Accommodation of Unrepeatable GE Through Adequate Testing

The solution to accommodating unrepeatable GE is to test adequately *within a target mega-environment*, i.e., to test at a sufficiently large number of locations in a sufficiently large number of years with sufficiently large number of replicates so as to sufficiently sample the environments and to achieve a sufficiently high heritability as defined in Equation 7 or Equation 8. It is obvious that more replicates, more locations, and more years will lead to a higher heritability. The solution to identify widely adapted cultivars (within a meg-environment) is to “test widely” (Troyer, 1996). However, each additional replicate, location, or year involves considerable cost. As a compromise between selection reliability and test cost, the concept “adequate test” was proposed and defined (Yan et al., 2015; Yan, 2016). The terms “adequate testing,” “optimum testing,” and “minimum testing” are used interchangeably in this article to indicate that a minimum level of testing in terms of years, locations, and replicates must be conducted to achieve sufficiently reliable selection. When tested inadequately, the selection intensity must be lowered according to the achieved heritability, to prevent superior genotypes from being mistakenly discarded. The “optimum” level of replicates, locations, or years was defined as one to achieve a heritability of 0.75, based on examining a heritability response curve (Yan et al., 2015; Figure 2 this article). However, Cobb et al. (2019) suggested that a heritability of 0.5 was sufficient for reliable selection of the best 10 individuals to be used to start the next breeding cycle.

### Optimum Number of Years

Based on Equation 7 and assuming neglectable GL and experimental error or unlimited number of locations and replicates, the minimum number of years required to achieve a heritability of 0.75 can be estimated by

(15) $$\begin{array}{l} y_{min}=\text{max}\left[1,\;3\left(\frac{\sigma_{GY}^{2}}{\sigma_{G}^{2}}\right)\right] \end{array}$$

For example, based on the yield data from three-year spans of Quebec provincial oat tests, the estimated minimum number of years to achieve a heritability of 0.75 was from 1.2 to 6.3 and averaged 3.2 (Table 2), while the officially required number of years to register a cultivar is three. So, the requirement for three years of testing was adequate and appropriate in general. More years of testing were required for the 2016–2018 and the 2018–2020 (Table 2) spans due to reduced genetic variability and therefore achieved heritability.

**Table 2:** The minimum number of years (*y*_*min*_) required to achieve a heritability (*H*) of 0.75 estimated on the yield data of three-year spans from the Quebec provincial oat registration trials.

**Three-year span**	**No. of genotypes**	** $\sigma_{G}^{2}$ **	** $\sigma_{GY}^{2}$ **	** $\sigma_{GLY}^{2}$ **	$\sigma_{P}^{2}$	**H**	***y***_***min***_
2013–2015	26	0.63	0.25	0.50	0.71	0.88	1.2
2014–2016	23	0.70	0.34	0.58	0.81	0.86	1.5
2015–2017	27	0.48	0.53	0.73	0.65	0.73	3.3
2016–2018	27	0.30	0.64	0.80	0.52	0.59	6.3
2017–2019	27	0.52	0.49	0.70	0.68	0.76	2.8
2019–2020	30	0.29	0.42	0.64	0.43	0.68	4.3
Mean							3.2

### Optimum Number of Locations

Yearly multi-location trials are usually balanced as the same set of genotypes are tested at all locations. Therefore, it is convenient to use yearly data to estimate the number of locations required for adequate testing. Assuming an infinite number of replicates or negligible experimental error, the heritability within a year (Equation 10) can be reduced to

(16) $$\begin{array}{l} H_{rl,max}=\frac{\sigma_{G}^{2}}{\sigma_{G}^{2}+\frac{\sigma_{GL}^{2}}{l}} \end{array}$$

where *H*_*rl,max*_ is the maximum achievable within-year heritability (Yan, 2021). Based on this equation, the minimum number of locations required to achieve a heritability of 0.75 can be estimated by (Yan et al., 2015; Yan, 2021)

(17) $$\begin{array}{l} l_{min}=\text{max}\left[1,\;3\left(\frac{\sigma_{GL}^{2}}{\sigma_{G}^{2}}\right)\right] \end{array}$$

The minimum number of locations so estimated is expected to differ with the year. Therefore, it should be estimated for a number of years to achieve a good understanding on the required number of test locations for a target mega-environment (Yan et al., 2015). Presented in Table 2 are the estimated yearly minimum number of locations based on the 2013–2019 Quebec provincial oat trial data for the two mega-environments as well as for the undivided Quebec oat growing region. When estimated for the undivided region, the mean number was 8.4, in comparison to the actual number of locations of 9.6. Thus, the number of locations used was more than adequate in most years.

Interestingly, when estimated for each mega-environment, the estimated minimum number was ~one location more than that actually used (7.3 vs. 5.9 for ME1 and 4.6 vs. 3.7 for ME2). Thus, even though there is a clear mega-environment differentiation, the trials in one mega-environment still provided useful information to selection for the other, because the two mega-environments were positively correlated (Figure 7). In contrast, the southern vs. northern oat mega-environments in eastern Canada were uncorrelated, and as a result, the total required number of locations was smaller when estimated separately for each mega-environment than that when estimated for the undivided region (Yan et al., 2015). In a Canada-wide study, the southern vs. northern mega-environments in eastern Canada were designated as ME1 and ME2, respectively, while the two Quebec mage-environments in Figure 4 or Figure 5 were designated as ME2a and ME2b (Yan et al., 2021). Given the results in Table 3, cultivar recommendation for each of the two Quebec mega-environments should consider performance both within the mega-environment and across the whole region, as shown in Figure 7.

**Table 3:** The estimated minimum number of locations in comparison to that actually used for the Quebec provincial oat trials.

	**The whole region**		**ME1^a^**		**ME2^a^**		**ME1 + ME2**
**Year**	**Actual**	**Estimated**	**Actual**	**Estimated**	**Actual**	**Estimated**	**Estimated^b^**
2013	8	11.2	5	5.7	3	4.6	10.3
2014	9	6.8	6	4.6	3	2.4	7.0
2015	10	6.8	6	9.6	4	1.5	11.1
2016	10	5.9	6	3.4	4	8.3	11.7
2017	10	8.2	6	8.8	4	1.4	10.2
2018	10	7.4	6	6.9	4	6.1	13.0
2019	10	12.5	6	12.4	4	7.7	20.1
Mean	9.6	8.4	5.9	7.3	3.7	4.6	11.9

a *See Figure 4 or Figure 5 for the definition of mega-environment 1 (ME1) and mega-environment 2 (ME2);*

b *The estimated number for ME1 + ME2 is the sum of the estimated number for ME1 and that for ME2*.

### Optimum Number of Replicates

Several classic studies investigated the optimum numbers of years, seasons, test locations, and replicates for the allocation of a fixed number of plots or fund according to the relative magnitudes of various variance components (Sprague and Federer, 1951; Hanson and Brim, 1963; Wricke and Weber, 1986; Swallow and Wehner, 1989). Conclusions from this “resource allocation” approach inevitably led to the suggestion to maximize the number of locations and/or years and to minimize the number of replicates (i.e., to use a single replicate) (McCann et al., 2012; Schmidt et al., 2018). However, this conclusion applies only when it is possible to increase the number of locations and/or years. For a breeding program or a regional crop recommendation committee, yield trials are conducted every year at a more or less fixed number of locations. Researchers need to know the minimum or optimum number of replicates under this scenario. To answer this question, an equation was derived from the definition of heritability on the single trial basis, in the form of Equations 15 and 17 (Yan et al., 2015). More recently, another equation was developed for estimating the optimum number of replicates in a multi-location context (Yan, 2021):

(18) $$\begin{array}{l} r_{l}=\text{max}\left[1,3\left(\frac{\sigma_{\varepsilon }^{2}}{\sigma_{G}^{2}}\right)\left(\frac{H_{rl,max}}{l}\right)\right] \end{array}$$

where *r*_*l*_ is the optimum number of replicates given the number of locations *l*, and *H*_*rl, max*_ is as defined in Equation 16. Equation 18 shows that the required number of replicates is determined by the relative magnitude of experimental error variance, $\frac{\sigma_{\varepsilon }^{2}}{\sigma_{G}^{2}}$, and is modified by the number of locations in a non-linear manner, because an increase in the number of locations also improves *H*_*rl, max*_ (Equation 16). Applying this equation to the yield data of some oat trials conducted across Canada, it was determined that two replicates would suffice to identify the highest yielding oat cultivars (Yan, 2021). Applying this equation to the 2015–2019 yield data of barley, oat, spring wheat, and winter wheat trials conducted in Ontario also led to the conclusion that two replicates would suffice (Yan et al., 2000). It is recommended that similar analysis be conducted for other crops and regions. Regional variety trials are usually conducted with three or four replicates. Reducing the number of replicates to two, if supported, can substantially reduce the evaluation cost or allow more genotypes to be evaluated with the same cost.

Importantly, reliable estimation of the various variances are a prerequisite to accurate estimation of the optimum number of years, optimum number of locations, and optimum number of replicates for adequate testing (Arief et al., 2015).

### Adjust for Spatial Variation

The discussions on optimum testing and optimum replication above assumed that the field and management are uniform within each trial. However, spatial variation within trials has been recognized as a major source of experimental error. Traditionally it is controlled by blocking, i.e., dividing a replicate into blocks, such as the so-called incomplete blocks design (R.A. Fisher, from Street, 1990). This is referred to “dealing with spatial variation by design.” In the last three decades, spatial analysis and adjustment becomes an increasingly popular research subject and a routine practice in the analysis of crop variety trials. The use of spatial analysis makes experimental design more flexible.

In a variety evaluation trial, *g* genotypes are usually allocated into a rectangular field of *b* rows (blocks) and *c* column (plots). The observed value in a plot, *Y*_*ij*_, is, therefore, combined effects of the row, the column, the genotype, and the experimental error:

(19) $$\begin{array}{l} Y_{ij}=\mu +row_{i}+col_{j}+g_{k}+\varepsilon_{ij}, \end{array}$$

**Figure 8:**
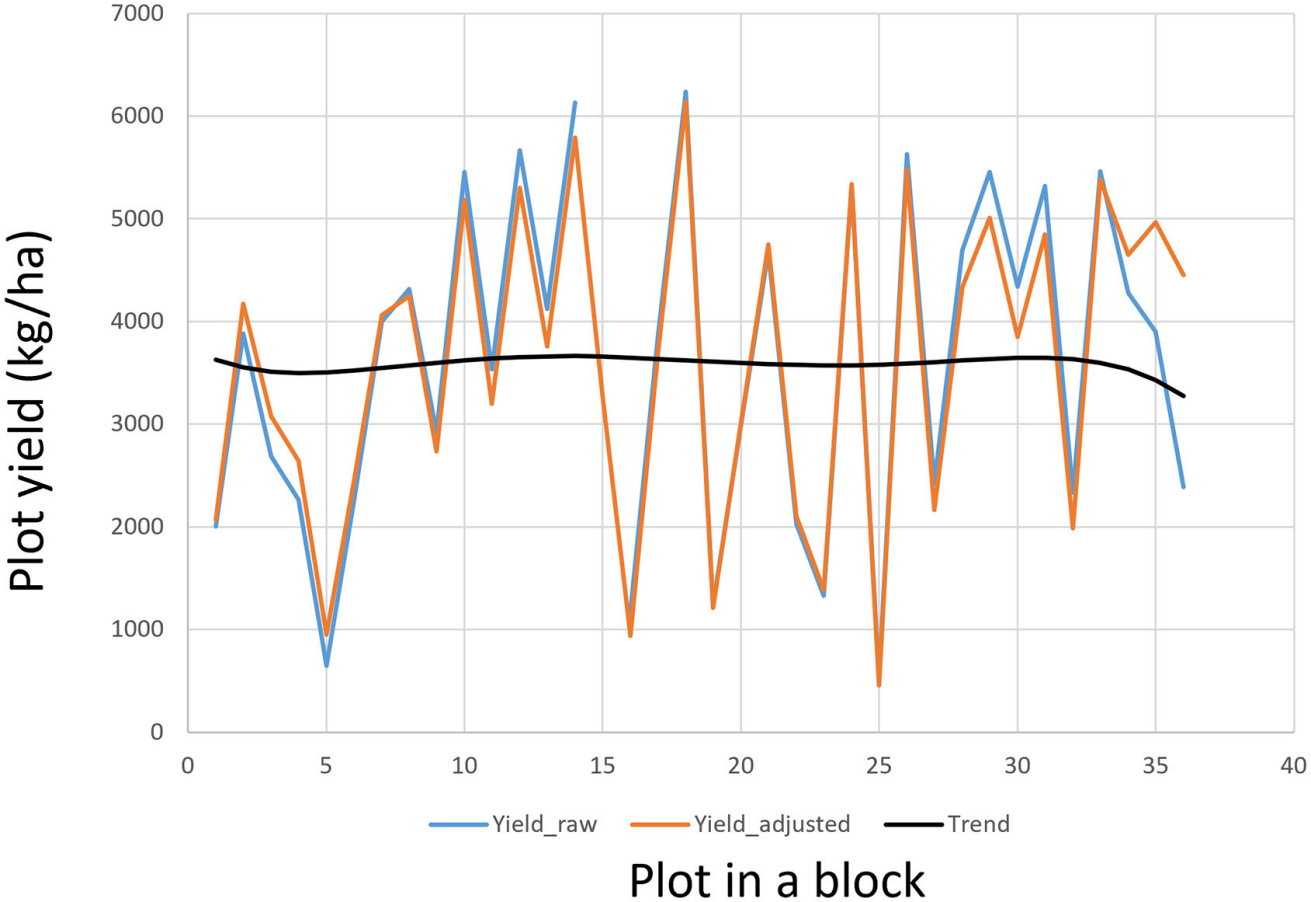
An example to show the plot values within a block as adjusted according to the field trend modeled by a polynomial regression.

μ being the mean of the trial. The effects of rows and columns can be modeled by various spatial analysis techniques (Cullis and Gleeson, 1991; Gilmour et al., 1997; Cullis et al., 1998; Burgueño et al., 2000; Qiao et al., 2000; Yang et al., 2004). Spatial analysis is a within-trial analysis so it is also referred to “local analysis” (Kempton et al., 1994; Grondona et al., 1996). A straightforward and intuitive approach is to use a polynomial regression to model any trend across the plots within each block (Yan, 2014), which is routinely used in the Ottawa oat breeding program. The order of the polynomial regression can be set according to the block size of the block. An iterative procedure can be used to adjust the *Y*_*ij*_ values so as to minimize the experimental error. Adjusted genotypic values are then calculated from the adjusted plot values at the final iteration. Spatial adjustment based on polynomial regression usually leads to reduced trial coefficient of variation and increased trial heritability (Yan, 2014). The plot values, and thus the genotypic values, will not be altered if no spatial trend is found. An example of spatial trend adjustment for a block of 36 plots in an oat trial conducted in Ontario in 2019 is presented in Figure 8. This procedure can also be used to fill missing plot values.

### Genomic Selection: To Replace Multi-Environment Evaluation With Multi-Model Prediction

Some researchers believe that genomic selection will eventually replace breeders' visual selection and even alter the role of yield trials in making selection and recommendation decisions (Heffner et al., 2009; Jannink et al., 2010). Indeed, encouraging results of genomic selection have started to emerge as advanced genotyping, bioinformatics, and genomic modeling procedures have become available (e.g., Tinker et al., 2016; Bekele et al., 2018). The confidence on genomic selection comes from two aspects. First, genome-wide markers can sufficiently capture the genotypic variability of a relevant breeding population tested in a relevant environment (i.e., a trial at a location in a year). That is, the genetic variability of the breeding population observed in the trial can be accurately captured by a genomic model. Second, genomic models can be developed for a large number of trials that sufficiently to fully represent the target mega-environment. Assuming *m* models have been developed from *m* trials covering multiple locations and years, then predicting the performance of a breeding population using *m* models would be equivalent to testing the breeding population in *m* trials. This represents a great advantage of genomic selection over conventional selection because in a practical breeding program, it is impossible to test a large breeding population in replicated trials, let alone at multiple locations in multiple years. Assuming a genotype by model two-way table of predictions for a breeding population, the achievable heritability with genomic selection, *H*_*m*_, can be estimated by

(20) $$\begin{array}{l} H_{m}=\frac{\sigma_{G}^{2}}{\sigma_{G}^{2}+\frac{\sigma_{\text{GM}}^{2}}{m}} \end{array}$$

where $\sigma_{\text{GM}}^{2}$ is the variance for genotype by model interaction. As *m* increases, the achievable heritability with genomic selection and hence selection reliability can become much higher than what is achievable by conventional selection (Yan et al., 2019b). From this viewpoint, genomic selection is potentially a much more effective approach to dealing with unrepeatable GE.

On the other hand, yield trials aiming at genomic model development are large and expensive; it remains a question as to when model development can be considered complete and such trials are no longer needed (Yan et al., 2019b). If the year factor is completely random, then genomic model development may be considered complete at some point when sufficient data (years and locations) have been obtained. However, if there is a trend in climatic change, data from recent years would be more relevant for predicting future-year performances, and the trials must continue. It is also a question whether routinely conducted yield trials with a limited number of entries can be used to replace the large-scale trials for genomic model development or refinement, although it was so suggested (Heffner et al., 2009). Cost efficiency will continue to be a determinant factor to the application of genomic selection in plant breeding.

## Selecting for Mean Performance and Selecting Against Instability

Superior cultivars must demonstrate high and stable yield cross the target mega-environment. Cultivars yielded well in some environments but poorly in others, relative to other cultivars, are said to be unstable and undesirable as they can cause unbearable losses to growers. Various stability indices have been developed in order to quantify instability. Lin et al. (1986) reviewed nine stability or instability parameters and classified them into four groups. More parameters were proposed after that (e.g., Huehn, 1990). The initial idea of stability analysis was to select against unstable genotypes rather than to select for stable genotypes. This idea was somehow twisted to treating a stability index as a positive trait, which caused confusion among researchers. It would be less confusing to call these stability indices as instability indices. The large number of indices are also confusing to practical breeders. The purpose of this section is to reinstall the original idea of stability analysis and to clear up the confusions.

First, a stability index should reflect a genotype's susceptibility to GE, because it is GE that caused its unstable performance across environments. The numerous stability parameters may be classified according to its composition in terms of G, E, and GE. In the classification of Lin et al. (1986), stability indices in Group B involve GE only; they are suitable instability parameters. Indices in group A involve both E and GE; they are not suitable parameters because GE is confounded with E. The linear regression coefficient *b* against E in Eberhart and Russell (1966) (Group C) is a genotype's response to E; its usefulness depends on how well the linear regression fits the data (Lin et al., 1986), which is usually poor (Zobel et al., 1988). When the fit is sufficiently good, *b* = 1 means stable, *b* > 1 means good performance in high-yielding environments, and *b* < 1 means good performance in low-yielding environments (Ceccarelli, 1989). Deviation from the linear regression (Group D) is merely a measure of the goodness of fit of the linear regression and is not a useful measure of stability.

Second, stability analysis is a concept of selection within a mega-environment. So, it should be conducted within mega-environments rather than across mega-environments, unless the mega-environments are highly correlated. In such cases, the mega-environments should be merged and treated as a single mega-environment.

Third, a stability index representing GE must be used in combination with the mean performance (G), thus the term G+GE or GGE (Yan et al., 2000, 2007). High stability (less GE) is desirable only when combined with high mean performance. High stability is least desirable when combined with low mean yield because it means consistently low yielding (Yan et al., 2007). Parameters or procedures combining both G and GE include the superiority index of Lin and Binns (1988, 1994) and the stability index of Kang (1993). In addition, several graphical methods also combines G and GE. These include the AMMI1 biplot (Zobel et al., 1988) and the AEA view of the GGE biplot (Figure 6; Yan, 2001). Zobel et al. (1988) showed that AMMI analysis was superior to the joint regression of Eberhart and Russell (1966) and Alwala et al. (2010) concluded GGE biplot analysis to be a better platform than the joint regression. There is some debate on whether AMMI or GGE is a better approach in analyzing yield trial data (Gauch, 2006; Yan et al., 2007; Gauch et al., 2008; Yan, 2011). Many studies compared AMMI and GGE biplot analysis and concluded that GGE biplot analysis was superior (e.g., Badu-Apraku et al., 2012; Amira et al., 2013; Hoyos-Villegas et al., 2016; Oliveira et al., 2019). AMMI analysis was advocated as a means to separate “signal” (true GE) from “noise” (error) in GE and a means to use the GE-signal to adjust the genotypic means (Gauch and Zobel, 1988; Gauch, 2013). AMMI1 or AMMI2 (i.e., main effects plus the first one or two principal components of GE) is often found the best AMMI model, and the AMMI1 biplot is often used as a visual tool for genotype evaluation (Gauch, 2006). Unfortunately, the AMMI1 biplot is not an effective graphical presentation of G + GE because G is often masked by the much larger E in it, because its G and GE axes are in different units, and because it does not have the inner-product property of a true biplot (Yan, 2011). Similar to AMMI analysis, GGE biplot analysis can also be viewed as a means to separate signal from noise. In a two-dimensional GGE biplot such as that in Figure 6, the first two principal components are considered as signals and the higher dimensions as noise. This GGE biplot displays the amount of G+GE in between that of AMMI1 and AMMI2; so, it should be close to the best model in most cases. In the AEA view of the GGE biplot (Figure 6), the GGE-mean axis represents the GE-adjusted genotypic means, while the GGE-stability axis represents the genotypes' susceptibility to unrepeatable GE (instability). Both axes pass through the biplot origin and are perpendicular to each other, meaning that they are independent parameters. Thus, the AEA view of the GGE biplot is a convenient tool for visual analysis of genotype-by-environment data and for visual selection for mean performance and against instability.

Finally, “test adequately” is much more important than any stability analysis. When tested adequately, genotypes with high mean performance should also be genotypes that are relatively stable, because it is not possible for a highly unstable genotype to achieve very high mean performance. However, when not tested adequately, as indicated by a low heritability across locations and years, neither the estimated mean nor the estimated stability is reliable, and a low selection intensity or culling rate must be applied. In such cases, selection should be mainly on mean performance, rather than on stability. Instead, effort should be made to understand the causes of the instability for a high-yielding genotype. For example, severe lodging may be the reason for its low yield in a severely lodged trial. If severe lodging rarely occurs in the target region, then the genotype is expected to show good mean yield and stability when tested adequately; if lodging is a common yield-limiting factor in the target region, then the genotype is expected to have low mean yield when tested adequately.

## Selection for Multiple Traits

While geneticists can focus on a single trait and ignore others, breeders must deal with multiple traits. In addition to high yield, which is always the most important breeding objective, a cultivar must meet a minimum requirement for each and every trait that is important to the relevant growers, processors, and end-users. In fact, the greatest challenge in plant breeding is to combine all desirable traits in a single genotype, because key breeding objectives are often adversely associated, due to either genetic linkage or pleiotropy (e.g., Tanksley, 1983; Yan and Wallace, 1995; Asins, 2002; Cooper et al., 2009; Hao et al., 2014; Crespo-Herrera et al., 2016). Strategies for multi-trait selection include independent culling and index selection (Simmonds and Smartt, 1999; Yan and Frégeau-Reid, 2008; Yan, 2014). Independent culling is to cull all genotypes that fail to meet the minimum requirement for any breeding objective, because such genotypes will not be accepted as cultivars. Index selection is to rank genotypes based on an index that is composed to reflect the perceived economic values of the genotypes. Independent culling can be implemented at all stages in the breeding cycle but it is more important in the early breeding stages when multi-location yield trials are not possible. Index selection is mainly implemented in the yield trial stage, at which all important traits can be determined. For cultivar development-oriented genomic selection, both independent culling and index selection should be conducted.

### Independent Culling

Independent culling is important to ensure that selected high yielding genotypes will be accepted by growers and end-users; it is also an effective approach to reduce the breeding population size safely and speedily. Assume that *t* is the number of independently inherited breeding objectives, each with a heritability $h_{k}^{2}$, with *k* = 1, …*t*. If $h_{k}^{2}$ is used as the culling rate for trait *k*, then the joint culling rate would be:

(21) $$\begin{array}{l} h_{t}^{2}=1-\underset{1}{\overset{t}{\prod }}\left(1-h_{k}^{2}\right) \end{array}$$

and the number of genotypes must be retained to ensure that the best genotype is selected is:

(22) $$\begin{array}{l} N=n\left(1-h_{t}^{2}\right),or \end{array}$$

$$\begin{array}{l} N/n=1-h_{t}^{2}, \end{array}$$

N/n being the proportion of the breeding population that must be retained. For example, assume the culling rate for each of five traits is 0.3, then, according to Equation 21, the joint culling rate would be 0.83, and the retaining rate would be 0.17 or 17%. Therefore, a large proportion of the population can be safely culled by independent culling if multiple traits are considered, even though the heritability or culling rate is low for each trait. This explains the effectiveness of visual selection (culling) by an experienced breeder, who can visualize and select on many traits simultaneously.

Genomic selection for oat yield in eastern Canada proves effective (Bekele, Tinker, and Yan, unpublished results); it should also be effective for other traits that are more simply inherited than yield. Therefore, independent culling based on genomic models is expected to be more accurate than visual selection by even the most experienced breeder. If the traits under consideration are positively correlated, the overall culling rate would be lower than when they are independent; the overall culling rate would be higher if the traits are negatively correlated, which is often the case. The overall culling rate can be much higher if some of the target traits are simply inherited and less affected by GE and experimental error, for example, oil content in oat (Hizbai et al., 2012; Yan et al., 2016).

### GYT (Genotype by Yield^*^trait) Analysis

A large portion of the genotypes that survived independent culling should be qualified as a cultivar if they are sufficiently high yielding. Therefore, the focus of selection following independent culling should be on yield although other target traits should also be considered. Selection based on a selection index is the common method for selection on multiple breeding objectives (note but: not any traits). Here, the GYT (genotype by yield^*^trait) analysis (Yan and Frégeau-Reid, 2018; Yan et al., 2019a) is recommended over the traditional index selection.

In traditional index selection, the superiority of genotype *i*, *P*_*i*_, is calculated as

(23) $$\begin{array}{l} P_{i}=w_{0}y_{i}+\overset{t}{\underset{j=1}{\sum }}\left(w_{j}x_{ij}\right), \end{array}$$

where *t* is the number of breeding objectives that are to be selected in addition to yield, *y*_*i*_ is the standardized yield for genotype *i*, *w*_0_ is the weight assigned for yield, *w*_*j*_ is the weight assigned for trait *j*, and *x*_*ij*_ is the standardized value of genotype *i* for trait *j*.

In the GYT approach, the superiority of a genotype may be presented as

(24) $$\begin{array}{l} P_{i}=y_{i}\overset{t}{\underset{j=1}{\sum }}\left(w_{j}x_{ij}\right). \end{array}$$

The selection index for a genotype is usually presented as the standardized value of *P*_*i*_. The difference between the traditional selection index (Equation 23) and the GYT approach (Equation 24) follows. In traditional index selection, the weight for a trait other than yield is a fixed value for all genotypes, while in the GYT approach it varies with the yield level of each genotype. In traditional index selection the emphasis is on the levels of the traits; in the GYT approach it is on the levels of yield-trait combinations. The GYT concept is better in reflecting the economic value of a trait. For example, superior lodging resistance (or high protein) has little value in a low yielding genotype but it is highly valuable in a high yielding genotype. Consequently, based on the traditional selection index, a low yielding genotype may be ranked the highest due to its superior levels in other traits; such genotypes will not be accepted as cultivars by growers, however (Yan et al., 2019a). This problem can be prevented with the GYT approach, as the genotypes ranked highest will always have high yield levels.

**Figure 9:**
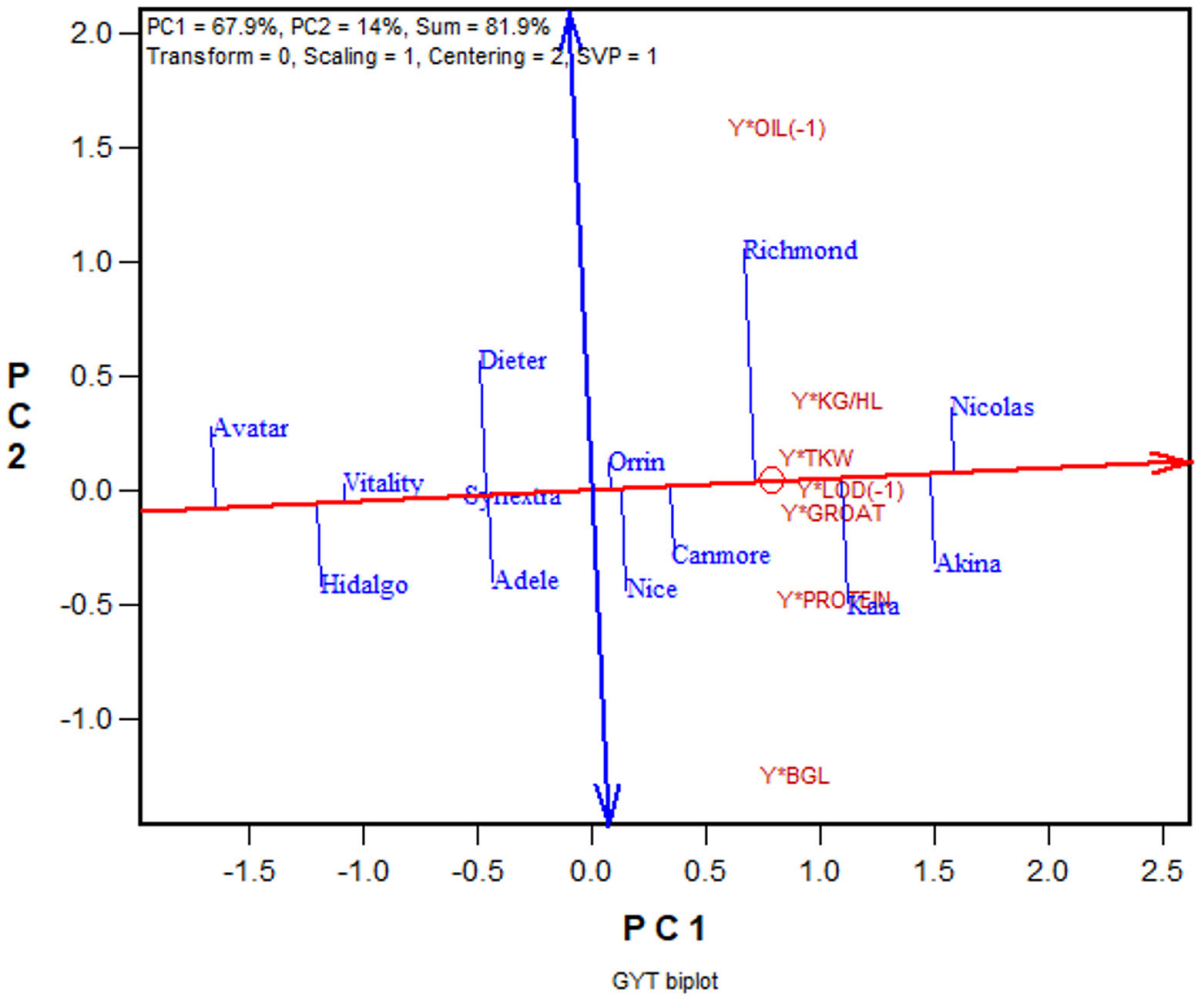
GYT biplot to display the yield-trait combinations of 13 oat cultivars tested in the 2013–2019 Quebec provincial oat trials. The biplot was based on singular value decomposition of yield-trait combination standardized data (“Centering = 2, Scaling = 1”). The red line with a single arrow is the average yield-trait axis, the arrow pointing to higher GYT index. The blue line with arrows on both ends indicate contrasting trait profiles of the genotypes. For example, it showed Richmond to be strong yield-oil combination but weak in yield-β-glucan combination, while Kara had the opposite trait profile to that of Richmond.

Another advantage of the GYT approach is that the superiority and the trait profiles of the genotypes can be visually investigated in a biplot, referred to as GYT biplot (Yan and Frégeau-Reid, 2018; Yan et al., 2019a,b). As an example, the mean values of the 13 oat cultivars for eight important traits from the 2013–2019 Quebec oat trials are presented in Table 4, ranked by their GYT index. The steps to construct a GYT biplot follow. First, standardize the genotype by trait table for each trait. Second, multiply yield with each trait to form a genotype by yield-trait combination two-way table. Third, subject the weighted genotype by yield-trait two-way able to singular value decomposition to obtain the principal components (PC). Fourth, multiply each of the yield-trait combination PC scores with the assigned weight. And finally, construct a biplot using the genotypic and trait combination scores of the first two principal components based on the yield-trait combination preserving singular value partition. Note that for milling oat a higher value is more desirable for all the traits listed in Table 4 except lodging and oil content. For these two traits a smaller value is more desirable; they were therefore given a weight of “−1.” The information contained in Table 4 can be visualized in a GYT biplot (Figure 9). The biplot clearly shows the rank of the genotypes in their GYT index, i.e., Nicolas > Akina > Kara > Richmond >…> Avatar. Thus, Nicolas and Akina should be selected and recommended to the Quebec oat growers without hesitation; they are in fact the most important two cultivars in Quebec. The biplot also shows the trait profiles of the genotypes. For example, it shows that Richmond is superior in having a low oil content but inferior in having a low β-glucan content. In fact, all cultivars placed above the red line (the GYT index axis) have relatively low oil and low β-glucan whereas the opposite is true for cultivars placed below the GYT index axis. “Y^*^Oil(−1)” and “Y^*^Lodging(−1)” indicate that oil content and lodging score were given a weight of “−1” because high oil content and high lodging are undesirable for milling oat (Figure 9).

**Table 4:** Mean trait values of 13 oat cultivars tested in the 2013–2019 Quebec provincial oat trials and their GYT (Genotype by Yield^*^Trait) index.

**Genotype**	**Traits and weights**								
	**Yield (kg ha** ^**−1**^ **)**	**β-glucan** **(%)**	**Groat (%)**	**Oil** **(%)**	**Protein (%)**	**Test weight** **(kg hl** ^**−1**^ **)**	**1000-Kernel Weight (g)**	**Lodging** **(0**–**9)**	**GYT Index**
		1	1	−1	1	1	1	−1	
Nicolas	5,948	4.3	73.9	6.1	13.2	53.4	35.8	2.9	1.3
Akina	5,853	4.8	72.7	7.1	13.4	52.3	38.0	2.4	1.2
Kara	5,680	4.7	71.9	8.0	14.0	54.0	38.1	2.0	0.9
Richmond	5,631	3.8	71.7	5.4	12.6	55.1	39.2	3.0	0.5
Canmore	5,447	4.6	71.6	7.6	14.2	54.9	39.5	3.3	0.3
Nice	5,559	4.4	72.6	8.4	13.5	53.3	38.5	3.9	0.1
Orrin	5,468	4.4	71.1	6.9	13.3	54.2	38.5	3.5	0.1
Adele	5,354	4.6	75.3	8.4	12.8	54.0	38.7	4.5	−0.3
Dieter	5,183	4.1	73.4	5.7	14.0	54.3	38.9	3.6	−0.4
Synextra	5,171	4.3	72.0	7.3	14.9	56.0	37.3	3.7	−0.4
Vitality	5,049	4.0	75.7	7.9	13.6	53.9	40.3	3.9	−0.9
Hidalgo	5,158	4.7	74.3	8.0	13.1	53.1	34.5	4.0	−0.9
Avatar	5,060	3.9	74.8	7.9	12.2	56.6	36.2	4.9	−1.3

The GYT biplot approach has been adopted in multi-trait selection for various crops (Boureima and Abdoua, 2019; de Oliveira et al., 2019; Hamid et al., 2019; Mohammadi, 2019; Gouveia et al., 2020; Mahmoud et al., 2020; Merrick et al., 2020; Badu-Apraku et al., 2021; Sofi et al., 2021; Tsenov et al., 2021; Xu et al., 2021).

## Conclusions

Plant breeding plays a key role in meeting the increasing need for food, fiber, health, and comfort and in combating the adverse impacts of the changing climate. Plant breeding consists of two stages: breeding population development and progeny selection. For cultivar development, population development is more important than progeny selection but has largely been neglected in the literature. Hence, a “complete breeder's equation” was presented, which contains three key parameters: the population mean, the population variability, and the achieved heritability under the multi-location, multi-year framework. The value of a breeding population is measured by both the population mean and the population variability. For progeny selection, the key is to improve the heritability, i.e., selection reliability. Three aspects were identified to improve heritability: utilizing repeatable GE through mega-environment analysis, accommodating unrepeatable GE by adequate testing, and adequate replication and adjusting for spatial variation. Procedures for mega-environment analysis include GGE + GGL biplot analysis and LG biplot analysis. Adequate testing includes estimation and use of an optimum number of years, locations, and replicates. Cultivar evaluation within a mega-environment should select for mean performance and select against instability, with GGE biplot analysis being a preferred graphical method. A stability index is meaningful only when combined with high mean yield. Adequate testing is more important than any stability analysis. Last but not least, cultivar development must consider multiple traits; both independent culling and index selection are essential. GYT biplot analysis is a preferred method for index selection. In addition, genomic selection is an alternative and potentially more effective approach in all stages and aspects of cultivar development if reliable models are developed and if it can be done cost-efficiently.

## Data Availability Statement

The original contributions presented in the study are included in the article/supplementary materials, further inquiries can be directed to the corresponding author.

## Author Contributions

The author confirms being the sole contributor of this work and has approved it for publication.

## Conflict of Interest

The author declares that the research was conducted in the absence of any commercial or financial relationships that could be construed as a potential conflict of interest.

## Publisher's Note

All claims expressed in this article are solely those of the authors and do not necessarily represent those of their affiliated organizations, or those of the publisher, the editors and the reviewers. Any product that may be evaluated in this article, or claim that may be made by its manufacturer, is not guaranteed or endorsed by the publisher.

## References

[B1] Alwala S. Kwolek T. McPherson M. Pellow J. Meyer D. (2010). A comprehensive comparison between Eberhart and Russell joint regression and GGE biplot analyses to identify stable and high yielding maize hybrids. Field Crops Res. 119, 225–230. 10.1016/j.fcr.2010.07.010

[B2] Amira J. O. Ojo D. K. Ariyo O. J. Oduwaye O. A. Ayo-Vaughan M. A. (2013). Relative discriminating powers of GGE and AMMI models in the selection of tropical soybean genotypes. Afr. Crop Sci. J. 21, 67–73.

[B3] Annicchiarico P. (2021). Breeding gain from exploitation of regional adaptation: an Alfalfa case study. Crop Sci. 61, 2254–2271. 10.1002/csc2.20423

[B4] Arief V. N. DeLacy I. H. Crossa J. Payne T. Singh R. Braun H. J.. (2015). Evaluating testing strategies for plant breeding field trials: redesigning a CIMMYT international wheat nursery. Crop Sci. 55, 164–177. 10.2135/cropsci2014.06.0415

[B5] Asins M. J. (2002). Present and future of quantitative trait locus analysis in plant breeding. Plant Breed. 121, 281–291. 10.1046/j.1439-0523.2002.730285.x20210691

[B6] Atlin G. N. Baker R. J. McRae K. B. Lu X. (2000). Selection response in subdivided target regions. Crop Sci. 40, 7–13. 10.2135/cropsci2000.4017

[B7] Atlin G. N. Kleinknecht K. Singh K. P. Piepho H. P. (2011). Managing genotype x environment interaction in plant breeding programs: a selection theory approach. J. Indian Soc. Agricu. Statistics 65, 237–247.

[B8] Badu-Apraku B. Bankole F. A. Ajayo B. S. Fakorede M. A. B. Akinwale R. O. Talabi A. O.. (2021). Identification of early and extra-early maturing tropical maize inbred lines resistant to *Exserohilum turcicum* in sub-Saharan Africa. Crop Protect. 139:105386. 10.1016/j.cropro.2020.105386 33390640 PMC7649949

[B9] Badu-Apraku B. Oyekunle M. Obeng-Antwi K. Osuman A. S. Ado S. G. Coulibay N.. (2012). Performance of extra-early maize cultivars based on GGE biplot and AMMI analysis. J. Agric. Sci. 150:473. 10.1017/S0021859611000761

[B10] Baum M. Lagudah E. S. Appels R. (1992). Wide crosses in cereals. Annu. Rev. Plant Biol. 43, 117–143. 10.1146/annurev.pp.43.060192.001001

[B11] Bekele W. A. Wight C. P. Chao S. Howarth C. J. Tinker N. A. (2018). Haplotype-based genotyping-by-sequencing in oat genome research. Plant Biotechnol. J. 16, 1452–1463. 10.1111/pbi.12888 29345800 PMC6041447

[B12] Boureima S. Abdoua Y. A. O. U. (2019). Genotype by yield^*^trait combination biplot approach to evaluate sesame genotypes on multiple traits basis. Turk. J. Field Crops 24, 237–244. 10.17557/tjfc.655165

[B13] Burgueño J. Cadena A. Crossa J. Banziger M. Gilmour A. R. Cullis B. (2000). User's Guide for Spatial Analysis of Field Variety Trials Using ASREML. Texcoco: CIMMYT.

[B14] Ceccarelli S. (1989). Wide adaptation: how wide? Euphytica 40, 197–205.

[B15] Cobb J. N. Juma R. U. Biswas P. S. Arbelaez J. D. Rutkoski J. Atlin G.. (2019). Enhancing the rate of genetic gain in public-sector plant breeding programs: lessons from the breeder's equation. Theor. Appl. Genet. 132, 627–645. 10.1007/s00122-019-03317-0 30824972 PMC6439161

[B16] Comstock R. E. Moll R. H. (1963). Genotype-environment interactions. Stat. Gen. Plant Breed. 982, 164–196.

[B17] Cooper M. van Eeuwijk F. A. Hammer G. L. Podlich D. W. Messina C. (2009). Modeling QTL for complex traits: detection and context for plant breeding. Curr. Opin. Plant Biol. 12, 231–240. 10.1016/j.pbi.2009.01.006 19282235

[B18] Cooper M. Voss-Fels K. P. Messina C. D. Tang T. Hammer G. L. (2021). Tackling G × E × M interactions to close on-farm yield-gaps: creating novel pathways for crop improvement by predicting contributions of genetics and management to crop productivity. Theor. Appl. Genet. 134, 1625–1644. 10.1007/s00122-021-03812-3 33738512 PMC8206060

[B19] Crespo-Herrera L. A. Velu G. Singh R. P. (2016). Quantitative trait loci mapping reveals pleiotropic effect for grain iron and zinc concentrations in wheat. Ann. Appl. Biol. 169, 27–35. 10.1111/aab.12276

[B20] Cullis B. Gogel B. Verbyla A. Thompson R. (1998). Spatial analysis of multi-environment early generation variety trials. Biometrics 54, 1–18. 10.2307/2533991

[B21] Cullis B. R. Gleeson A. C. (1991). Spatial analysis of field experiments-an extension to two dimensions. Biometrics 47, 1449–1460. 10.2307/2532398

[B22] de Oliveira T. R. A. de Amaral Gravina G. de Moura Rocha M. de Alcântara Neto F. da Cruz D. P. de Oliveira G. H. F.. (2019). GYT biplot analysis: a new approach for cowpea line selection. J. Exp. Agric. Int. 41, 1–9. 10.9734/jeai/2019/v41i530408

[B23] DeLacy I. H. Basford K. E. Cooper M. Bull J. K. McLaren C. G. (1996). Analysis of multienvironment trialsa historical perspective, in Plant Adaptation and Crop Improvement, eds Cooper M. Hammer G. L. (Wallingford: IRRI/CABI), 39124.

[B24] Duvick D. N. (1996). Plant breeding, an evolutionary concept. Crop Sci. 36, 539–548. 10.2135/cropsci1996.0011183X003600030001x

[B25] Eberhart S. A. (1970). Factors affecting efficiencies of breeding methods. Afr. Soils 15, 655–680.

[B26] Eberhart S. T. Russell W. A. (1966). Stability parameters for comparing varieties 1. Crop Sci. 6, 36–40. 10.2135/cropsci1966.0011183X000600010011x

[B27] Fehr W. (1991). Principles of Cultivar Development: Theory and Technique. Stuttgart: Macmillian Publishing Company.

[B28] Gabriel K. R. (1971). The biplot graphic display of matrices with application to principal component analysis. Biometrika 58, 453–467. 10.1093/biomet/58.3.453

[B29] Gauch G. H. Jr. Zobel R. W. (1997). Identifying mega- environments and targeting genotypes. Crop Sci. 37, 311–326.

[B30] Gauch H. G. Jr. Piepho H. P. Annicchiarico P. (2008). Statistical analysis of yield trials by AMMI and GGE: further considerations. Crop Sci. 48, 866–889. 10.2135/cropsci2007.09.0513

[B31] Gauch H. G. Jr. (2006). Statistical analysis of yield trials by AMMI and GGE. Crop Sci. 46, 1488–1500. 10.2135/cropsci2005.07-019327323051

[B32] Gauch H. G. Jr. (2013). A simple protocol for AMMI analysis of yield trials. Crop Sci. 53, 1860–1869. 10.2135/cropsci2013.04.0241

[B33] Gauch H. G. Jr. Zobel R. W. (1988). Predictive and postdictive success of statistical analyses of yield trials. Theor. Appl. Genet. 76, 1–10. 24231975 10.1007/BF00288824

[B34] Gilmour A. R. Cullis B. R. Verbyla A. P. (1997). Accounting for natural and extraneous variation in the analysis of field experiments. J. Agric. Biol. Environ. Stat. 2, 269–293. 10.2307/1400446

[B35] Gilmour A. R. Thompson R. Cullis B. R. (1995). Average information REML: an efficient algorithm for variance parameter estimation in linear mixed models. Biometrics 51, 1440–1450. 10.2307/2533274

[B36] Goddard M. E. Hayes B. J. (2007). Genomic selection. J. Anim. Breed. Genet. 124, 323–330. 10.1111/j.1439-0388.2007.00702.x 18076469

[B37] Gouveia B. T. Barrios S. C. L. do Valle C. B. da Costa Gomes R. Machado W. K. R. de Sousa Bueno Filho J. S.. (2020). Selection strategies for increasing the yield of high nutritional value leaf mass in Urochloa hybrids. Euphytica 216, 1–12. 10.1007/s10681-020-2574-3

[B38] Grondona M. O. Crossa J. Fox P. N. Pfeiffer W. H. (1996). Analysis of variety yield trials using two-dimensional separable ARIMA processes. Biometrics 52, 763–770. 10.2307/2532916

[B39] Hamid A. E. Aglan M. A. Hussein E. (2019). Modified method for the analysis of genotype by trait (Gt) biplot as a selection criterion in wheat under water stress conditions. Egypt. J. Agron. 41, 293–312. 10.21608/agro.2019.16580.1177

[B40] Hanson W. D. Brim C. A. (1963). Optimal allocation of test material for two-stage testing with an application to evaluation of soybean lines. Crop Sci. 3, 43–49.

[B41] Hao Y. Velu G. Peña R. J. Singh S. Singh R. P. (2014). Genetic loci associated with high grain zinc concentration and pleiotropic effect on kernel weight in wheat (*Triticum aestivum* L.). Mol. Breed. 34, 1893–1902. 10.1007/s11032-014-0147-7

[B42] Heffner E. L. Sorrells M. E. Jannink J. L. (2009). Genomic selection for crop improvement. Crop Sci. 49, 1–12. 10.2135/cropsci2008.08.0512

[B43] Hizbai B. T. Gardner K. M. Wight C. P. Dhanda R. K. Molnar S. J. Johnson D.. (2012). Quantitative trait loci affecting oil content, oil composition, and other agronomically important traits in oat. Plant Genome 5, 164–175. 10.3835/plantgenome2012.07.0015

[B44] Hoyos-Villegas V. Wright E. M. Kelly J. D. (2016). GGE biplot analysis of yield associations with root traits in a Mesoamerican bean diversity panel. Crop Sci. 56, 1081–1094. 10.2135/cropsci2015.10.0609

[B45] Huehn M. (1990). Nonparametric measures of phenotypic stability. Part 2: applications. Euphytica 47, 195–201.

[B46] Jannink J. L. Lorenz A. J. Iwata H. (2010). Genomic selection in plant breeding: from theory to practice. Brief. Funct. Genomics 9, 166–177. 10.1093/bfgp/elq001 20156985

[B47] Kang M. S. (1993). Simultaneous selection for yield and stability in crop performance trials: Consequences for growers. Agron. J. 85, 754–757. 10.2134/agronj1993.00021962008500030042x

[B48] Kempton R. A. Seraphin J. C. Sword A. M. (1994). Statistical analysis of two-dimensional variation in variety yield trials. J. Agric. Sci. 122, 335–342. 10.1017/S0021859600067253

[B49] Kharkwal M. C. Pandey R. N. Pawar S. E. (2004). Mutation breeding for crop improvement, in Plant Breeding, eds Jain H. K. Kharkwal M. C. (Dordrecht: Springer), 601–645. 10.1007/978-94-007-1040-5_26

[B50] Lin C. S. Binns M. R. (1988). A superiority measure of cultivar performance for cultivar × location data. Canad. J. Plant Sci. 68, 193–198. 10.4141/cjps88-01824213184

[B51] Lin C. S. Binns M. R. (1994). Concepts and methods for analyzing regional trial data for cultivar and location selection. Plant Breed. Rev. 12, 271–297. 10.1002/9780470650493.ch10

[B52] Lin C. S. Binns M. R. Lefkovitch L. P. (1986). Stability analysis: where do we stand? Crop Sci. 26, 894–900.

[B53] Ma F. Xu Y. Ma Z. Li L. An D. (2018). Genome-wide association and validation of key loci for yield-related traits in wheat founder parent Xiaoyan 6. Mol. Breed. 38, 1–15. 10.1007/s11032-018-0837-7

[B54] Mahmoud M. W. Hussein E. Aboelkassem K. M. Ibrahim H. E. (2020). Graphical presentation of some peanut genotypes by comparing two patterns of biplot analysis. J. Plant Produ. 11, 697–705. 10.21608/jpp.2020.112895

[B55] McCann L. C. Bethke P. C. Casler M. D. Simon P. W. (2012). Allocation of experimental resources to minimize the variance of genotype mean chip color and tuber composition. Crop Sci. 52, 1475–1481. 10.2135/cropsci2011.07.0392

[B56] Merrick L. F. Glover K. D. Yabwalo D. Byamukama E. (2020). Use of genotype by yield^*^trait (GYT) analysis to select hard red spring wheat with elevated performance for agronomic and disease resistance traits. Crop Breed. Genet. Genomics 2:e200009. 10.20900/cbgg20200009

[B57] Mohammadi R. (2019). Genotype by yield^*^ trait biplot for genotype evaluation and trait profiles in durum wheat. Cereal Res. Commun. 47, 541–551. 10.1556/0806.47.2019.32

[B58] Oliveira T. R. A. D. Carvalho H. W. L. D. Oliveira G. H. F. Costa E. F. N. Gravina G. D. A. Santos R. D. D.. (2019). Hybrid maize selection through GGE biplot analysis. Bragantia 78, 166–174. 10.1590/1678-4499.20170438

[B59] Piepho H. P. Büchse A. Emrich K. (2003). A hitchhiker's guide to mixed models for randomized experiments. J. Agron. Crop Sci. 189, 310–322. 10.1046/j.1439-037X.2003.00049.x

[B60] Qiao C. G. Basford K. E. DeLacy I. H. Cooper M. (2000). Evaluation of experimental designs and spatial analyses in wheat breeding trials. Theor. Appl. Genet. 100, 9–16. 10.1007/s001220050002

[B61] Rasmusson D. C. Phillips R. L. (1997). Plant breeding progress and genetic diversity from de novo variation and elevated epistasis. Crop Sci. 37, 303–310. 10.2135/cropsci1997.0011183X003700020001x

[B62] Rutkoski J. E. (2019). A practical guide to genetic gain. Adv. Agron. 157, 217–249. 10.1016/bs.agron.2019.05.001

[B63] Schmidt P. Möhring J. Koch R. J. Piepho H. P. (2018). More, larger, simpler: how comparable are on-farm and on-station trials for cultivar evaluation? Crop Sci. 58, 1508–1518. 10.2135/cropsci2017.09.0555

[B64] Shu Q. Y. Forster B. P. Nakagawa H. Nakagawa H. (2012). Plant Mutation Breeding and Biotechnology. Oxford: CAB International. 10.1079/9781780640853.0000

[B65] Simmonds N. Smartt J. (1999). Principles of Crop Improvement, 2nd Edn. Oxford: Blackwell Science Ltd. Press.

[B66] Singh R. K. Chaudhary B. D. (1977). Biometrical Methods in Quantitative Genetic Analysis. Ludhiana: Kalyani.

[B67] Sofi P. A. Saba I. Ara A. Rehman K. (2021). Comparative efficiency of GY^*^T approach over GT approach in genotypic selection in multiple trait evaluations: case study of common bean (*Phaseolus vulgaris*) grown under temperate Himalayan conditions. Agric. Res. 1–9. 10.1007/s40003-021-00577-5

[B68] Sprague G. F. Federer W. T. (1951). A comparison of variance components in corn yield trials. II. Error, year × variety, location × variety and variety components. Agron. J. 43, 535–541.

[B69] Street D. (1990). Fisher's contributions to agricultural statistics. Biometrics 46, 937–945. 10.2307/2532439

[B70] Swallow W. H. Wehner T. C. (1989). Optimum allocation of plots to years, seasons, locations, and replications, and its application to once-over-harvest cucumber trials. Euphytica 43, 59–68. 10.1007/BF00037897

[B71] Tanksley S. D. (1983). Molecular markers in plant breeding. Plant Mol. Biol. Rep. 1, 3–8.

[B72] Tinker N. A. Bekele W. A. Hattori J. (2016). Haplotag: software for haplotype-based genotyping-by-sequencing analysis. G3 (Bethesda) 6, 857–873. 10.1534/g3.115.024596 26818073 PMC4825656

[B73] Troyer A. F. (1996). Breeding widely adapted, popular maize hybrids. Euphytica 92, 163–174. 10.1007/BF00022842

[B74] Tsenov N. Gubatov T. Yanchev I. (2021). Genotype selection for grain yield and quality based on multiple traits of common wheat (*Triticum aestivum* L.). Cereal Res. Commun. 49, 119–124. 10.1007/s42976-020-00080-7

[B75] van Harten A. M. (1998). Mutation Breeding: Theory and Practical Applications. Cambridge: Cambridge University Press.

[B76] Wang L. Zhu G. Johnson W. Kher M. (2018). Three new approaches to genomic selection. Plant Breed. 137, 673–681. 10.1111/pbr.12640

[B77] Wricke G. Weber E. (1986). Quantitative Genetics and Selection in Plant Breeding. Berlin, NY: Walter de Gruyter

[B78] Xu N. Y. Zhao S. Q. Zhang F. Fu X. Q. Yang X. N. Qiao Y. T. Sun S. X. (2021). Retrospective evaluation of cotton varieties nationally registered for the Northwest Inland cotton growing regions based on GYT biplot analysis. Acta Agron. Sinica 47, 660–671. 10.3724/SP.J.1006.2021.04135

[B79] Yan W. (2001). GGEbiplot—A Windows application for graphical analysis of multienvironment trial data and other types of two-way data. Agron. J. 93, 1111–1118. 10.2134/agronj2001.9351111x

[B80] Yan W. (2002). Singular-value partitioning in biplot analysis of multienvironment trial data. Agron. J. 94, 990–996. 10.2134/agronj2002.0990

[B81] Yan W. (2011). GGE biplot vs. AMMI graphs for genotype-by-environment data analysis. J. Indin Soc. Agric. Stat. 65, 181–193.

[B82] Yan W. (2013). Biplot analysis of incomplete two-way data. Crop Sci. 53, 48–57. 10.2135/cropsci2012.05.0301

[B83] Yan W. (2014). Crop Variety Trials: Data Management and Analysis. Hoboken, NJ: John Wiley & Sons. 10.1002/9781118688571

[B84] Yan W. (2015). Mega-environment analysis and test location evaluation based on unbalanced multiyear data. Crop Sci. 55, 113–122. 10.2135/cropsci2014.03.0203

[B85] Yan W. (2016). Analysis and handling of G × E in a practical breeding program. Crop Sci. 56, 2106–2118. 10.2135/cropsci2015.06.0336

[B86] Yan W. (2019). LG biplot: a graphical method for mega-environment investigation using existing crop variety trial data. Sci. Rep. 9, 1–8. 10.1038/s41598-019-43683-9 31073232 PMC6509248

[B87] Yan W. (2021). Estimation of the optimal number of replicates in crop variety trials. Front. Plant Sci. 11:590762. 10.3389/fpls.2020.590762 33519847 PMC7838102

[B88] Yan W. Frégeau-Reid J. (2008). Breeding line selection based on multiple traits. Crop Sci. 48, 417–423. 10.2135/cropsci2007.05.0254

[B89] Yan W. Frégeau-Reid J. (2018). Genotype by yield* trait (GYT) biplot: a novel approach for genotype selection based on multiple traits. Sci. Rep. 8:8242. 10.1038/s41598-018-26688-8 29844453 PMC5974279

[B90] Yan W. Frégeau-Reid J. Martin R. Pageau D. Mitchell-Fetch J. (2015). How many test locations and replications are needed in crop variety trials for a target region? Euphytica 202, 361–372.

[B91] Yan W. Frégeau-Reid J. Mountain N. Kobler J. (2019a). Genotype and management evaluation based on genotype by yield^*^trait (GYT) analysis. Crop Breed. Genet. Genomics 1:e190002. 10.20900/cbgg20190002

[B92] Yan W. Frégeau-Reid J. Pageau D. Martin R. (2016). Genotype-by-environment interaction and trait associations in two genetic populations of oat. Crop Sci. 56, 1136–1145. 10.2135/cropsci2015.11.0678

[B93] Yan W. Hunt L. A. Sheng Q. Szlavnics Z. (2000). Cultivar evaluation and mega environment investigation based on the GGE biplot. Crop Sci. 40, 597–605.

[B94] Yan W. Kang M. S. (2002). GGE Biplot Analysis: A Graphical Tool for Breeders, Geneticists, and Agronomists. CRC press.

[B95] Yan W. Kang M. S. Ma B. Woods S. Cornelius P. L. (2007). GGE biplot vs. AMMI analysis of genotype-by-environment data. Crop Sci. 47, 643–653. 10.2135/cropsci2006.06.0374

[B96] Yan W. Mitchell-Fetch J. Beattie A. Nilsen K. T. Pageau D. DeHaan B.. (2021). Oat mega-environments in Canada. Crop Sci. 61, 1143–1153. 10.1002/csc2.20426

[B97] Yan W. Pageau D. Frégeau-Reid J. Lajeunesse J. Goulet J. Durand J. Marois D. (2011). Oat mega-environments and test-locations in Quebec. Canad. J. Plant Sci. 91, 643–649. 10.4141/cjps10139

[B98] Yan W. Tinker N. A. (2005). An integrated biplot analysis system for displaying, interpreting, and exploring genotype × environment interaction. Crop Sci. 45, 1004–1016. 10.2135/cropsci2004.0076

[B99] Yan W. Tinker N. A. (2006). Biplot analysis of multi-environment trial data: principles and applications. Canad. J. Plant Sci. 86, 623–645. 10.4141/P05-169

[B100] Yan W. Tinker N. A. Bekele W. A. Mitchell-Fetch J. Frégeau-Reid J. (2019b). Theoretical unification and practical integration of conventional methods and genomic selection in plant breeding. Crop Breed. Genet. Genomics 1:e190003. 10.20900/cbgg20190003

[B101] Yan W. Wallace D. H. (1995). Breeding for negatively associated traits. Plant Breed. Rev. 13, 141–177. 10.1002/9780470650059.ch4

[B102] Yang R. C. Ye T. Z. Blade S. F. Bandara M. (2004). Efficiency of spatial analyses of field pea variety trials. Crop Sci. 44, 49–55. 10.2135/cropsci2004.0049

[B103] Zhao H. Z. Zhang H. F. Song Z. M. (1981). Several key issues in wheat breeding. Shaanxi Agric. Sci. 3, 1–8. In Chinese.

[B104] Zobel R. W. Wright M. J. Gauch H. G. Jr. (1988). Statistical analysis of a yield trial. Agron. J. 80, 388–393. 10.2134/agronj1988.00021962008000030002x

